# Self-reported aggressiveness does not follow facial masculinization in diverse South Asian populations

**DOI:** 10.1093/pnasnexus/pgag299

**Published:** 2026-09-04

**Authors:** Karel Kleisner, Zuzana Štěrbová, Vojtěch Fiala, Eliška Podgorná, Abhishikta Ghosh Roy, Banita Behera, Deepika Rani, Kewal Krishan, Stanzin Namga, Sanzida Khatun, Abdul Raheem Jesmil, Abdul Hameed, Viktor Černý

**Affiliations:** Department of Philosophy and History of Science, Faculty of Science, Charles University, Viničná 7, 128 44 Prague 2, Czech Republic; Department of Philosophy and History of Science, Faculty of Science, Charles University, Viničná 7, 128 44 Prague 2, Czech Republic; Department of Psychology, Faculty of Arts, Charles University, nám. Jana Palacha 1/2, 116 38 Prague 1, Czech Republic; Department of Philosophy and History of Science, Faculty of Science, Charles University, Viničná 7, 128 44 Prague 2, Czech Republic; Institute of Advanced Studies, Nicolaus Copernicus University in Toruń, Wileńska 4, 87-100 Toruń, Kuyavian-Pomeranian Voivodeship, Poland; Department of Natural Sciences and Archaeometry, Institute of Archaeology, Czech Academy of Sciences, Letenská 123/4, 118 00 Prague 1, Czech Republic; Department of Anthropology, Visva-Bharati, A Central University and an Institute of National Importance, Santiniketan, Birbhum District 731235, West Bengal, India; Anthropological Survey of India, Ministry of Culture, Government of India, 27 Jawaharlal Nehru Road, Kolkata 700016, West Bengal, India; Department of Anthropology, Panjab University, Chandigarh 160014, Chandigarh Union Territory, India; Department of Anthropology, Panjab University, Chandigarh 160014, Chandigarh Union Territory, India; Department of Geology, University of Jammu, Baba Saheb Ambedkar Road, Jammu 180006, Jammu and Kashmir, India; Department of Anatomy, Birat Medical College Teaching Hospital, Tankisinuwari, Morang 56613, Koshi Province, Nepal; Department of Archaeology, Ministry of Buddhasasana, Religious and Cultural Affairs, Sir Marcus Fernando Mawatha, Colombo 00700, Western Province, Sri Lanka; Department of Archaeology, Hazara University Mansehra, Khyber Pakhtunkhwa 21120, Pakistan; Department of Natural Sciences and Archaeometry, Institute of Archaeology, Czech Academy of Sciences, Letenská 123/4, 118 00 Prague 1, Czech Republic; Department of Anthropology and Human Genetics, Charles University, Viničná 7, 128 44 Prague 2, Czech Republic

**Keywords:** Bayesian modeling, cross-cultural variation, facial morphology, geometric morphometrics, human face, personality, sexual dimorphism, sexual selection

## Abstract

It has been suggested that sexually dimorphic facial traits are related to competitive behavior and aggression, but empirical evidence remains inconsistent and is based on a small number of populations and culturally homogeneous samples. We have combined geometric morphometric analyses with hierarchical Bayesian modeling of sixteen culturally and geographically diverse but understudied ethnic groups in order to test whether self-reported aggression covaries with facial sexual shape dimorphism within and between populations. Across populations, we found no consistent association between the individual level of facial sexual dimorphism and self-reported aggression in either sex. Although populations did differ in mean aggression levels, mean self-reported aggression covaried neither with the magnitude of sexual dimorphism nor with overall facial masculinization. Finally, sex differences in self-reported aggression did not reflect morphological sex differences. These findings challenge the generalizability of claims that aggression is encoded in the sexually dimorphic facial structure.

Significance Statement Masculine-looking faces are widely believed to signal aggressiveness, yet supporting evidence has been mixed and drawn mostly from limited, culturally homogeneous samples. We tested this assumption in 16 diverse and previously understudied populations from South Asia and Cabo Verde using geometric morphometrics and Bayesian modeling. Across both women and men, facial sexual dimorphism did not reliably predict self-reported aggression within populations. At the population level, average aggression also did not track facial masculinization or the degree of facial sex differences. These findings challenge the idea that aggressive disposition is biologically encoded in sexually dimorphic facial structure and suggest that common inferences from faces may reflect perceptual heuristics rather than valid behavioral information.

## Introduction

Humans tend to assess aggressive tendencies based on sexually dimorphic facial traits, and these inferences can in turn influence important social judgments (1–5). Nevertheless, it remains unclear to what extent facial morphology reliably reflects actual aggressive tendencies across populations (6, 7). By clarifying these relationships, we can shed light on both interindividual differences and broader social dynamics.

Self-reported aggression, as measured by the Buss–Perry Aggression Questionnaire (BPAQ; 8), is a stable personality trait that encompasses physical aggression (PHAGR), verbal aggression (VAGR), anger (ANGER), and hostility (HOST). The BPAQ shows robust correlations with act-based measures of aggression, especially in terms of direct aggressive acts aimed at same-sex opponents (*r* = 0.60 for PHAGR and *r* = 0.52 for VAGR; 9). Shorter questionnaires, such as the Brief Aggression Questionnaire, likewise show that aggressive tendencies can be assessed via self-reporting (10).

Existing research suggests that certain facial characteristics may provide cues to these dispositional tendencies. In particular, the facial width-to-height ratio (fWHR) has been linked to self-reported aggression and dominance in both men and women, with higher ratios predicting greater scores on the subscales of PHAGR and VAGR (11, 12). However, other studies, which used direct anthropometric measures (13) or standardized photographs (14, 15), found either weak or no associations between facial structure and self-reported aggressive tendencies. Overall, these findings suggest that while specific facial features may convey partial information about interindividual differences in self-reported aggression, the associations are modest at best.

While self-reports capture trait-level or dispositional aggression, behavioral measures assess actual aggressive acts in situational contexts. Research based on laboratory paradigms or naturalistic observations suggests that men with a higher fWHR display increased reactive aggression; this is reflected both in the results of the Point Subtraction Aggression Paradigm and in competitive sports contexts, for instance in success in hockey penalties (11). Meta-analyses have reported small but significant associations between fWHR and male aggression (6, 16), which may be moderated by social status (17), but a large-scale cross-cultural study which investigated the sexual dimorphism of fWHR found no consistent link to aggressive or criminal behavior (18). Similarly, no associations were found between fWHR and aggressive behavior measured in football players by fouls committed or yellow and red cards received (19). Kosinski (7) reevaluated fWHR in a large sample of 137,163 participants and found no substantial associations with behavioral traits, including aggression. Overall, it thus seems that while certain facial features may be associated with aggression (both as a trait and as a behavior), the effects tend to be small, inconsistent, and sample-dependent.

Human facial sexual dimorphism arises from androgen-dependent developmental processes which shape both the craniofacial bone structure and soft-tissue morphology; this process takes place most notably during puberty, when testosterone accelerates the growth of the mandible, brow ridge, and midface (20–22). Geometric morphometric analyses consistently show that the “masculine” facial shape, characterized by a relatively wider and longer lower face, more pronounced brow ridges, and a more robust mandible, occupies a specific and replicable region of a multidimensional shape space (23, 24). Importantly, several studies have demonstrated direct associations between hormonal markers and these shape dimensions: certain single nucleotide polymorphisms linked to circulating testosterone and sex hormone-binding globulin significantly predict variations of mandibular shape in large genomic samples (25), while higher levels of prenatal testosterone (measured in umbilical-cord blood) predict a more masculinized adult facial shape (22). But not all the proposed markers are reliably linked between hormones and morphology. For instance, the widely used fWHR often leads to just a low level of sexual dimorphism in adults (26). In some populations, fWHR is even higher in women than in men, which suggests the existence of population-specific dimorphism patterns (27). Moreover, once the size of viscerocranium is controlled for, shape differences on their own permit only modest accuracy of sex classification, which suggests that dimorphism often reflects size-related allometry rather than discrete shape differences (28). It seems, therefore, that while androgen-related developmental processes do contribute to sexually dimorphic facial morphology, empirical evidence points to various heterogeneous, population-dependent associations. This underscores the need for comprehensive geometric morphometric approaches rather than reliance on particular univariate indices.

In the present study, we have used a comprehensive dataset consisting of standardized facial images (frontal and profile) and BPAQ scores from 16 populations, mostly from South Asia. These populations are mostly understudied (they include communities with limited literacy and minimal prior participation in any research), which makes them particularly informative for testing the generalizability of associations between facial morphology and aggressiveness. By capturing variation across different genetic, cultural, and environmental contexts, we can evaluate whether previously reported links hold beyond the narrow, culturally homogeneous samples typical of prior research. We apply a rigorous analytical framework—a combination of geometric morphometrics with multilevel Bayesian modeling—to examine five specific research questions (Q1–5) related to both individual and population levels.

First (Q1), we investigate the individual-level validity of association between the face and aggression. We want to know whether individual sexually dimorphic facial traits reliably predict the same individual's self-reported aggression once the confounding effects of body mass index (BMI) and age are rigorously controlled for.

Second (Q2), extending our analysis to the population level, we draw on links between intense male–male competition and aggressive behavior predicted by the sexual selection theory (9, 29–31). In this context, we ask whether populations with higher mean total aggression (TAGR; used as a proxy of the intensity of intrasexual competition) exhibit more pronounced sexual shape dimorphism (SShD).

Third (Q3), assuming a shared physiological substrate where androgens drive both craniofacial growth and behavioral reactivity, we test whether populations characterized by a more masculinized average phenotype exhibit higher mean self-reported aggression.

Fourth (Q4), we examine the cross-domain consistency of sexual differentiation. In particular, we test whether populations with greater facial sexual dimorphism also exhibit greater differences between the sexes in self-reported aggression. This would enable us to determine whether physical and self-reported BPAQ sex differences are systematically associated or their mutual relationship fluctuates across populations.

Finally (Q5), extending our analysis from dimorphism to overall morphology, we employ multivariate shape regression to test whether self-reported aggression maps directly onto facial shape space. This should help us determine whether specific facial phenotypes reliably reflect aggressive tendencies on individual and population level.

## Materials and methods

### Population sampling and data collection

To ensure a representative cross-section of South Asian diversity, we have sampled populations across Pakistan, India, Nepal, Bangladesh, and Sri Lanka, thus encompassing distinct geographic regions, four large linguistic families (Indo-Aryan, Dravidian, Austroasiatic, and Tibeto-Burman), and various religious backgrounds (mainly Muslim, Hindu, and Buddhist). In Pakistan, the data were collected from the Kalash, Kashmiri, and Sindhi populations (all Indo-Aryan). In India, we have sampled in the Odisha state the Khond (Dravidian) and Gadaba peoples (Austroasiatic), in the Ladakh state from the Ladakhi and Tibetans (both Tibeto-Burman), and in North India, the Himachal Pradesh people (North Indians). In Nepal, we have sampled the Sherpa and Tamang people (both Tibeto-Burman) and the Chhetri and Tharu (both Indo-Aryan). In Bangladesh, we have sampled the Bengalis (Indo-Aryan), and in Sri Lanka, from the Sinhalese (Indo-Aryan) and the Tamil populations (Dravidian). In addition, we have sampled the population of Cabo Verde, which represents a genetic and geographic outgroup characterized by historical mixing of European and African populations. We took this step to see whether our findings can be generalized beyond Asian populations.

Recruitment was conducted in collaboration with researchers from local universities and research institutes who facilitated community engagement, explained the goals of the study in the participants’ native languages, and administered questionnaires. Sampling took place between May 2023 and December 2025 and followed a balanced design, with ∼100 individuals (50 men and 50 women) targeted per population.

All participants were asked to report their age (mean age = 37.76 years, SD = 15.58 years), ethnicity, and primary language. All in all, the final sample from all target countries includes the data of 742 women and 736 men (after the exclusion of outliers caused by measurement errors in the field). For a summary of the participants’ descriptive statistics, see Table S5.

The study was approved by the Ethics Committee of Charles University in Prague (approval no. 2023/06). The photographs were taken with the assistance of local researchers who carefully explained to participants the purpose of data collection and asked them to arrive for data collection at a designated place. Prior to data collection, and only after the local assistants confirmed that participants fully understood the process and what their consent involves, participants signed the informed consent form. Our initial aim was to have all participants complete questionnaires independently but since some populations included illiterate participants, trained research assistants in such cases read the items aloud and recorded participants’ responses (for questionnaire administration procedures across populations, see Table S1 and Section S1).

### The aggression questionnaire

Aggression was measured using the 29-item Buss–Perry Aggression Questionnaire (BPAQ; 8), which assesses PHAGR, VAGR, ANGER, and HOST. The TAGR score is then computed as the sum of all items. Responses were indicated on a Likert scale. In all but two populations the response format was the standard 1–5 scale (anchored with 1 = extremely uncharacteristic and 5 = extremely characteristic). In the Kashmiri and Kalash samples, we used a 1–7 format, whereby the responses were linearly rescaled to match the 5-point metric (see Section S2). The questionnaire was translated into all languages by native speakers. For descriptive statistics of the questionnaires, see Section S3 and Table S2.

**Figure 1:**
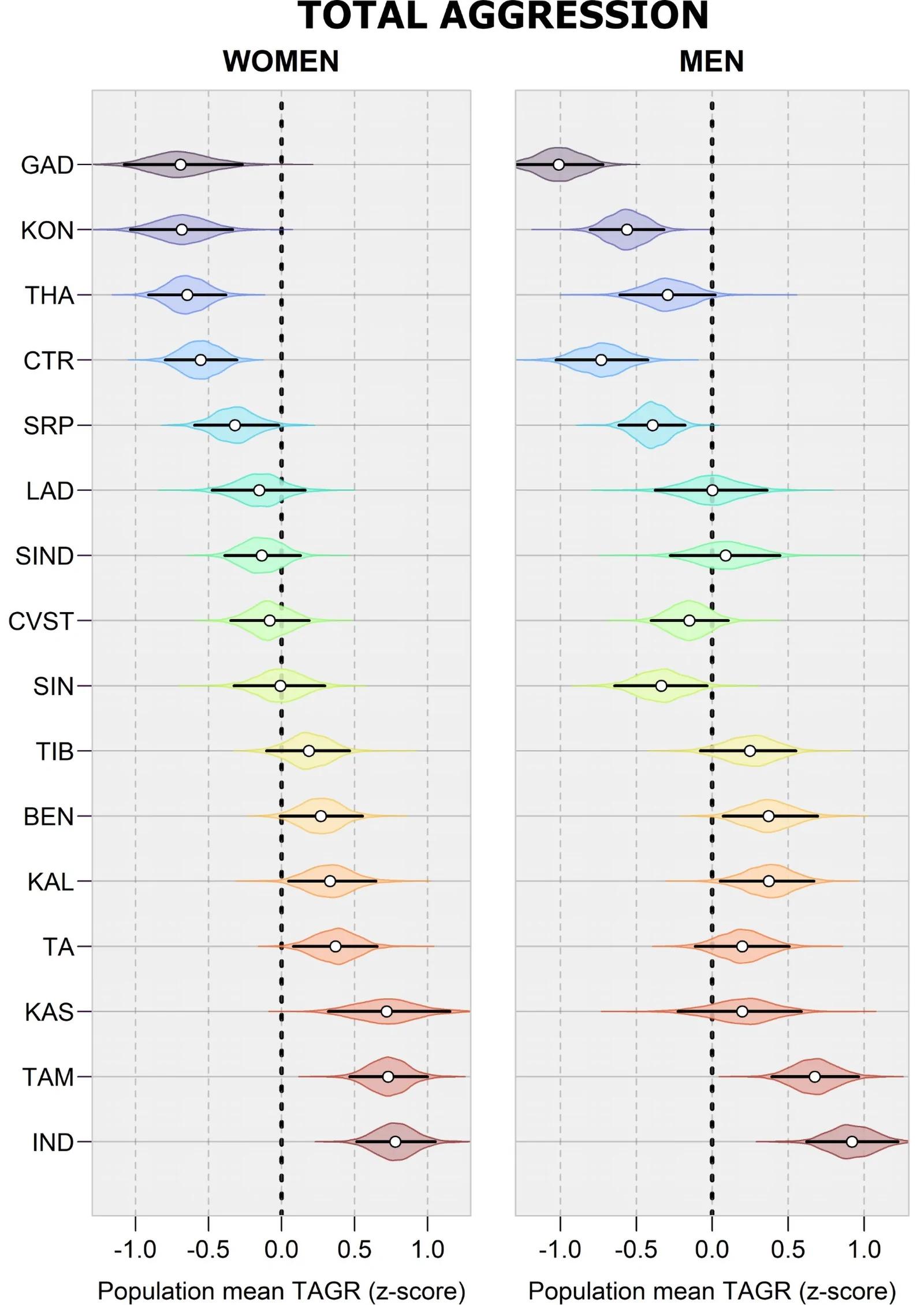
Population mean values of TAGR score expressed as z-scores (scaled across populations) for all populations, shown separately for women (left) and men (right). White points indicate population means, violin plots show the distribution of values within each population. The vertical dashed line marks the overall mean (*z* = 0). Population abbreviations (top to bottom on the left) correspond to the following groups: GAD, Gadaba; KON, Khond; THA, Tharu; CTR, Chhetri; SRP, Sherpa; LAD, Ladakhi; SIND, Sindhi; CVST, Cabo Verdeans; SIN, Sinhalese; TIB, Tibetans; BEN, Bengali; KAL, Kalash; TA, Tamang; KAS, Kashmiri; TAM, Tamil; IND, North Indians (Punjabi).

Missing data were imputed using person-mean substitution when less than 50% of items were missing; otherwise, the score was coded as missing (see Section S4 and Table S3). Cronbach's alpha was computed separately for each cultural group and each subscale using SPSS Version 31 (see Section S5). Reliability values for all groups are provided in Table S4. Populations with total scale alpha ≥0.70 were retained for main analyses (32). See Fig. 1 for a comparison of mean TAGR scores summarized by the sampled populations.

### Acquisition of photographs

All of the photographed individuals were instructed to gaze directly at the camera, maintain a neutral facial expression, and avoid any head tilting or other deviations from the Frankfort horizontal plane. All possible effort was taken to ensure uniform settings, especially in terms of preventing any photographic distortions between different individuals or datasets. Facial portraits were acquired following a standardized procedure (33). All photographs across all populations were taken by the same researcher (VČ) to ensure maximum procedural consistency. Images were taken with a full-frame camera Canon EOS 6D equipped with a Sigma lens with fixed focal length of 85 mm. Distance between the camera and the subject was set at 125 cm. We took several photographs, usually three or four, of the same person and then carefully selected those where the subject was best aligned with the vertical axis in the case of enfaces and with the horizontal axis in the case of profiles. Each facial photograph included a standardized grayscale card, which provided reference for color calibration, exposure adjustments, and white balance.

The photographs were taken in an uncompressed RAW format and later converted to JPEG files in the sRGB color space using Adobe Photoshop Lightroom 4. Colors and exposure were calibrated using grayscale and color cards consistently across all samples in the dataset.

### Application of landmarks and the Procrustes fit

To capture the variation of facial shape, we have defined 72 landmarks on each face from the frontal view; 36 of these were semilandmarks describing curves and outlines. For the profile view, we have defined 21 landmarks, including 6 semilandmarks. This process was implemented using the FaceDig 1.0 software for automated landmark placement (34). Furthermore, we have checked with principal component analysis for the presence of outliers in the dataset caused by landmark position errors. For definitions and locations of landmarks and semilandmarks, see our previous work (35–37).

Facial shape coordinates represented by landmarks and semilandmarks were then superimposed by Generalized Procrustes Analysis using the gpagen() function in the geomorph package in R (38, 39). Semilandmarks were slid along the tangent and their positions optimized by an algorithm that minimizes the bending energy between corresponding points.

### SShD and the masculinization index

SShD scores, which indicate the morphological level of individual development of sexually dimorphic traits, were computed using aligned shape coordinates that resulted from a joint Procrustes fit of female and male facial configurations. First, we have determined in the facial morphospace a vector connecting the sex-specific averages; this vector defined the shape differences between the men and women in our sample. Then we computed individual SShD scores by projecting each face onto a vector connecting the sex-specific averages (24, 40). Higher negative scores indicate a more feminine morphology, while higher positive scores indicate a more masculine facial structure. This process was repeated separately for each population.

To quantify overall facial masculinization at a population level, we used a sex-adjusted index derived from the global SShD axis. First, we computed global mean SShD scores separately for the males and the females. For each individual, we then calculated a sex-adjusted SShD score as that individual's SShD value minus the global mean of their sex. Finally, we have averaged these sex-adjusted scores across all individuals within each population. The resulting population-level index can thus be interpreted as the mean displacement of faces (both male and female) along the global SShD axis relative to the corresponding global sex-specific means, with higher values indicating populations whose faces are overall more masculinized.

### Bayesian multilevel modeling

#### Within-population Bayesian analyses (Q1)

We have analyzed the association between facial morphology and aggression using Bayesian multilevel regression implemented in the *brms* R package (41), which provides an interface to Stan's Hamiltonian Monte Carlo algorithms. All continuous variables—including profile and enface components of SShD, age, BMI, and aggression scores—were standardized within each population to allow for a direct comparison of effect sizes. Because previous work has indicated sex-specific relationships between the facial structure and behavioral traits, we estimated sex-specific intercepts and slopes. To do so, we have encoded all predictors in a sex-specific form, thus creating parallel regressors for women and for men. This coding scheme yielded separate parameters for each sex and avoided the interpretational complexities associated with interaction terms.

On top of that, participants were nested within national populations, so the models included sex-specific varying intercepts and slopes per each ethnic group. For each population, we have estimated a multivariate normal vector of random effects, thus allowing the influence of facial shape, age, and BMI to differ across populations while retaining partial pooling toward the overall mean.

To account for Galton's problem (42), we constructed a linguistic similarity matrix from the Automated Similarity Judgment Program (ASJP) lexical data (43) and used it to define the covariance structure of the ethnic-group-specific random effects in a Bayesian multiple regression, following Bürkner (44). The similarity matrix has been introduced using reversed Levenshtein distance normalized divided (LDND). Briefly, this distance corresponds to difference between two strings, expressed as the minimum number of single-character edits to get from one string to another, divided by the number of characters of the longer string (45). The resulting distance is subsequently divided by average Leveshtein distance among words that do not refer to the same concept to account for between-words’ similarities that appear by chance. If multiple synonyms were listed for the same English term, we used the first equivalent to prevent potential ambiguities in the ASJP database from altering the results.

First, we have fitted univariate Gaussian multilevel models for the TAGR score, calculated from BPAQ subscales: PHAGR, VAGR, ANGER, and HOST. TAGR score for participant *i* in population *j* was modeled as a linear combination of sex-specific intercepts, sex-specific effects of facial shape, age, and BMI, and vectors of population-level deviations in these variables, thus capturing between-population variability. Depending on the model specification, these population-level effects were either estimated using a conventional multilevel structure or modeled with a covariance structure informed by linguistic relatedness (LDND), allowing populations that are linguistically more similar to exhibit more similar parameter values. Besides the LDND-informed covariance structure, random effects were allowed to correlate freely, enabling the model to estimate whether populations which differ in mean aggression also differ systematically in the effects of morphological predictors (enface and profile SShD).

For overall inference, we have built a joint multivariate model that simultaneously estimated all four aggression subscales (PHAGR, VAGR, ANGER, and HOST) in a single hierarchical framework. This model had the same fixed- and random-effect structure as the univariate models but additionally allowed the residuals of the four outcomes to covary. This allowed us to capture shared variance between the dimensions of aggression that arise independently of SShD or demographic covariates, which in turn improved precision by borrowing statistical strength across related outcomes. The model was fitted using four Markov chains with extended warmup, an adapt-delta threshold of 0.98, and an increased tree depth to ensure stable sampling for this high-dimensional random effects structure.

Priors were chosen so as to be weakly regularizing; they follow the current recommendations for multilevel behavioral modeling (41). Regression coefficients were assigned normal priors centered at zero with moderate variance, residual standard deviations followed heavy-tailed Student-t distributions, and group-level standard deviations were assigned exponential priors, which favor modest between-population differences while permitting larger deviations where supported by the data. Correlation matrices for both the random effects and residuals were assigned LKJ priors with shape parameter 2; this setup slightly favors weaker correlations but does not unduly constrain the posteriors. Convergence was assessed through effective sample sizes, potential scale reduction statistics, and via visual inspection of sampling chains; all parameters reached R-hat values indistinguishable from 1.00.

Posterior inference is based on the means and 95% compatibility intervals and sex-specific effects are interpreted directly from the model's parameterization. For visualization, we have plotted posterior density estimates for fixed effects and, where relevant, we plotted also the distribution of between-population variance components.

#### Between-population Bayesian analyses (Q2–Q4)

As indicated above, our aim was to explore three population-level hypotheses: first (Q2), the notion that populations with higher mean total self-reported aggression (TAGR) exhibit stronger SShD in facial morphology (defined as the male–female difference); second (Q3), that populations with more masculinized facial morphology (quantified as the sex-adjusted mean SShD) exhibit higher mean TAGR; and third (Q4), that populations with greater sex differences in SShD also exhibit greater sex-level differences in TAGR.

Individual data were aggregated to the population level (N = 16). For each population, we have computed the mean TAGR from individual sex-specific TAGR means and the difference between the sexes (male TAGR–female TAGR). For SShD, we have computed—separately for profile and enface views—sex-specific population means and dimorphism (male SShD–female SShD). To construct facial masculinization index, we have first computed each individual's score on the global SShD axis derived from the pooled sample (i.e. with all populations joined into a single dataset prior to axis estimation). Overall facial masculinization was then expressed as a sex-specific mean-centered SShD score obtained by subtracting the global sex-specific mean (calculated across all individuals pooled over all populations) from each individual's SShD value.

Each hypothesis was evaluated using an independent Bayesian linear regression across populations in the form of *y* = *α* + *βx* + *ε*, where *ε* was normally distributed with mean 0 and variance *σ*^2^, and fitted in brms under a Gaussian likelihood. Responses were the population mean TAGR for Q2 and Q3 and TAGR sex difference for Q4; predictors were SShD sex-difference or sex-balanced SShD (profile and enface), and analogously for BMI or height measures. This yielded parallel sets of models. Predictors and outcomes were z-standardized within each model. We used weakly informative priors: *α*, *β* ∼ Normal(0, 1); *σ* ∼ Exponential(1). Models were run with four Markov chains of 2,000 iterations each and adapt delta set to 0.95. Inference focused on posterior estimates of *β* and their 95% credible intervals.

### Shape regressions (Q5)

Facial shape was quantified using geometric morphometrics based on Procrustes-aligned landmark configurations extracted from the frontal and profile facial views. Analyses were conducted separately for women and men. To assess associations between facial shape and self-reported aggression, we have fitted multivariate linear models using residual randomization permutation procedures (RRPP) implemented in the geomorph's procD.lm() function.

To disentangle individual level from population-level associations, TAGR was decomposed into a within-population component (W.TAGR; individual deviation from the population mean) and a between-population component (B.TAGR; population mean assigned to individuals). All predictors were standardized prior to analysis, and age and BMI were included as covariates.

We used two complementary modeling strategies. First, we tested global associations between facial shape, TAGR, and the population by fitting interaction models (TAGR × population). Second, we fitted within–between models of the form shape ∼W.TAGR + B.TAGR + age + BMI, separately for each sex and facial view, using RRPP with randomization of null-model residuals. As a robustness check, we have additionally entered the age and BMI as within-population centered covariates and constrained permutations within populations (blocked the RRPP) to control for the population structure.

### Data availability

The data underlying this article, together with the R code required to reproduce all analyses and figures, are available in the Open Science Framework repository (46) at https://osf.io/bvg98/. The deposit comprises the Procrustes-aligned landmark configurations, the individual SShD scores, the BPAQ item and scale scores, the demographic covariates entered into the models, the linguistic similarity matrix derived from the ASJP database, and the analysis scripts. The original facial photographs are not publicly redistributed, because the informed consent obtained from participants does not extend to the public release of identifiable facial images.

## Results

### Self-reported aggression does not reflect the individual masculine–feminine facial traits (Q1)

**Figure 2:**
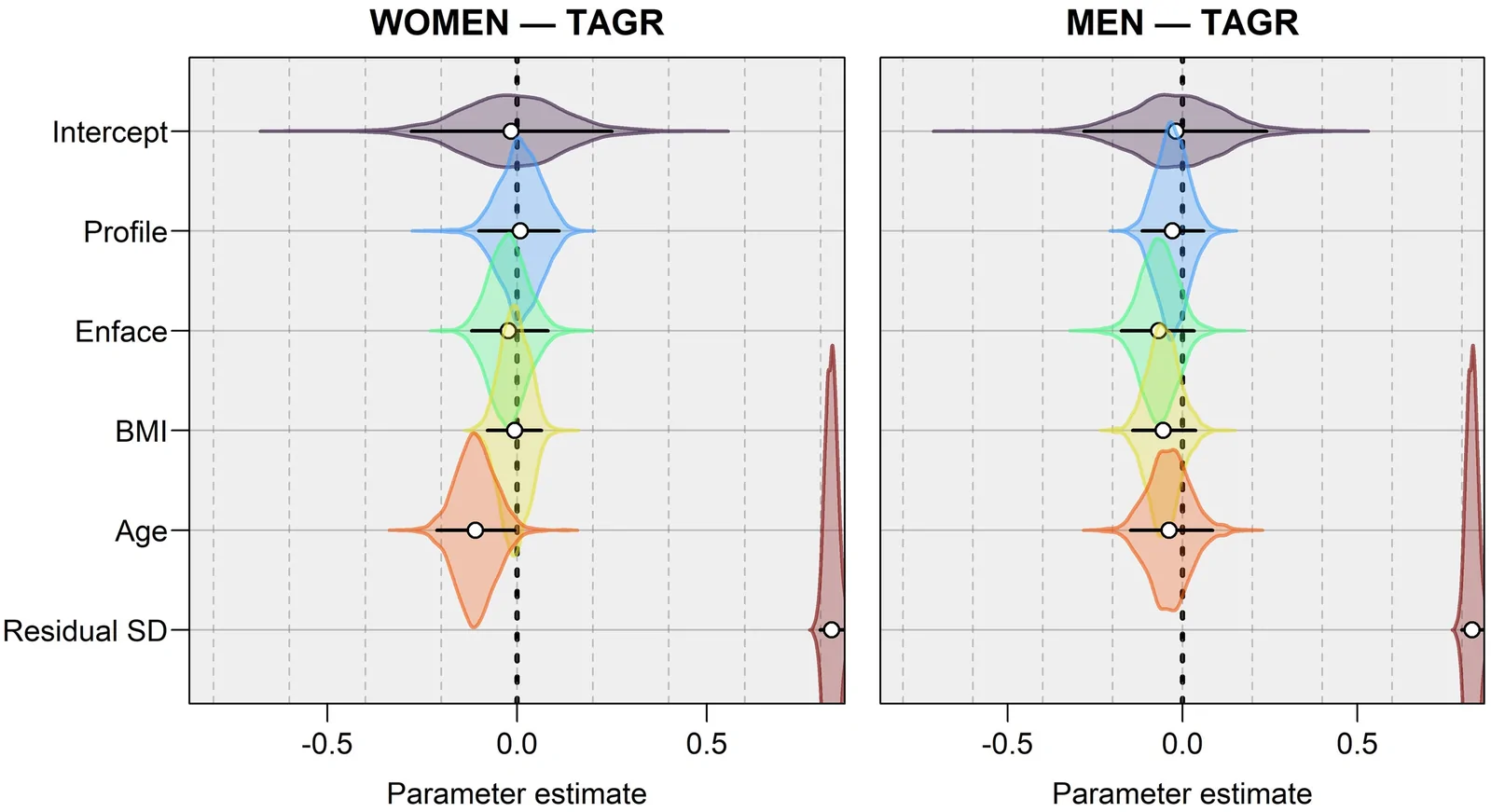
Density plots showing the posterior distributions of fixed-effect parameter estimates on a standardized scale from multilevel Bayesian models predicting total aggression score (TAGR). Model parameters include the model intercept and the following predictors: Profile, SShD derived from the profile face view, Enface, frontal face view SShD, BMI, body mass index; Age, age of the individual. The residual SD represents the remaining unexplained variation in TAGR. Black error bars indicate 95% credible intervals of the posterior distributions, circles denote posterior means. Distributions reflect uncertainty around parameter estimates after accounting for population-level structure and individual variation. For a full report on the model's predictions, see Table S6.

In the 16 populations we have examined, we found no evidence that SShD, BMI, or age have any substantial or systematic effects on BPAQ self-reports in either sex. In the case of model with TAGR as a single response (Fig. 2), posterior estimates for all fixed effects centered near zero and their credible intervals broadly overlapped zero. This indicates that neither profile nor enface SShD, or age, nor BMI reliably predict TAGR ratings for women or men. The only exception was a weak negative trend for male BMI, although the 95% credible interval included zero.

Importantly, introducing linguistic covariance as a proxy for population relatedness does not substantially change the model's predictive ability. Expected log predictive density is similar across parameterizations (range 1.7 elpd): models that combine the linguistic similarity matrix with independent varying effects (M2 and M3), the model that attributes all between-population variation to linguistic relatedness (M1), and the model that ignores the covariance altogether (M0) all perform comparably. None of these parameterizations alters the substantive conclusions reported below.

Despite the absence of strong global effects, the model revealed marked cross-national variability in the overall TAGR levels. Intercepts for women and men varied similarly across populations (SD = 0.52 and 0.53, respectively), which indicates substantial cultural heterogeneity in baseline evaluations. Slope variation for all predictors was comparatively small, although credible intervals indicated that populations differ modestly in how SShD and covariates relate to TAGR.

Residual variation remained high (*σ* = 0.83), indicating that the predictors we included explained only a small proportion of the total variance of TAGR. The only consistent population-level effect was a modest negative association between age and TAGR in women (*β* = −0.11, 95% CI [−0.21, −0.01]), while in men the corresponding effect was weaker and not credibly nonzero (*β* = −0.05, 95% CI [−0.18, 0.07]). Collectively, these results show that variation in TAGR is dominated by population-level differences rather than by consistent effects of SShD, BMI, or age.

**Figure 3:**
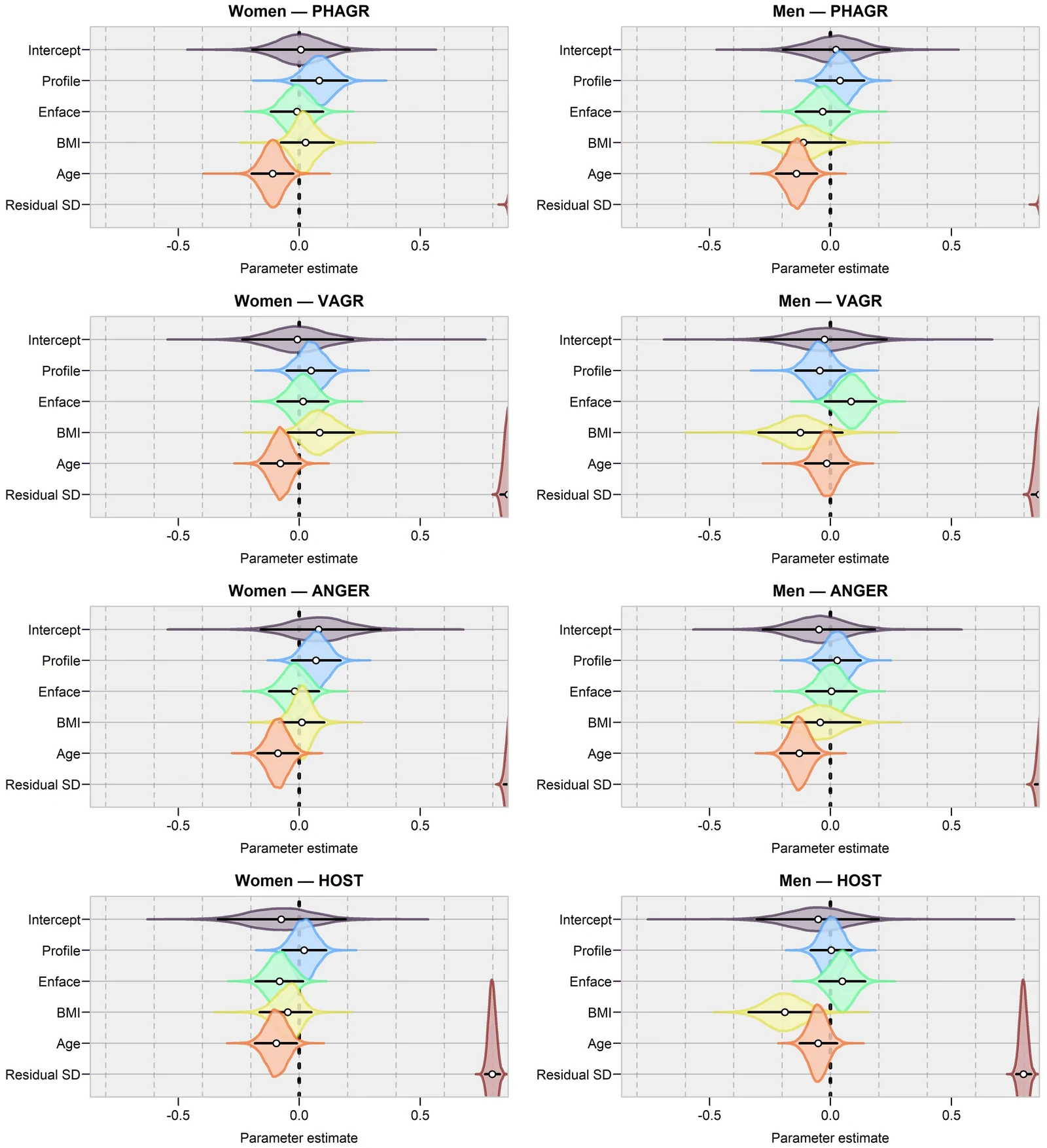
Density plots showing the posterior distributions of fixed-effect parameter estimates on a standardized scale from the multilevel Bayesian models that predict different domains of aggression: PHAGR, physical aggression; VAGR, verbal aggression; ANGER, anger; HOST, hostility. Aside from the Intercept (absolute term), the models include the following predictors: Profile, profile face view; SShD Enface, frontal face view SShD; BMI, body mass index; Age, age of individual. Black error bars indicate 95% credible intervals of the posterior distributions, circles denote posterior means. Distributions reflect uncertainty around parameter estimates after accounting for population-level structure and individual variation. See Table S7 for a full report of the model results.

In a joint multivariate model of the four BPAQ subscales, we have observed a comparable pattern (Fig. 3) where most slopes for SShD, BMI, and age remained close to zero, with only modest, more consistent negative associations of age with PHAGR and ANGER (*β* ≈ −0.10 to −0.12) and a weak negative association between male BMI and HOST (*β* ≈ −0.07). Residual variances were again substantial across subscales (*σ* ≈ 0.81–0.91) and the subscales were strongly intercorrelated at the residual level (*r* ≈ 0.41–0.52), which indicates that individual differences in the four facets of BPAQ are largely shared and remain mostly unexplained by the morphological and demographic predictors included in the model. Separate models which considered individual subscales independently as the sole dependent variable yielded similar (mostly null) results.

### Between-population comparisons show no association between self-reported aggression and facial dimorphism (Q2–Q4)

Further, we have conducted between-population analyses to test whether *population-level variation* in self-reported aggression covaries with facial SShD (Q2), overall facial masculinization (Q3), and the magnitude of sex differences (Q4). Populations with higher mean TAGR showed no larger SShD (male mean–female mean) in either the profile configuration (*β* = 0.32, 95% CI [−0.19, 0.84]) or the enface configuration (*β* = 0.32, 95% CI [−0.21, 0.84]). Likewise, populations with higher levels of TAGR did not exhibit overall more masculinized facial structure in either the profile (*β* = 0.36, 95% CI [−0.15, 0.84]) or the enface shape (*β* = −0.34, 95% CI [−0.86, 0.16]). Finally, populations with greater TAGR sex differences did not show greater SShD sex differences for either the profile (*β* = −0.08, 95% CI [−0.60, 0.44]) or the enface configuration (*β* = −0.15, 95% CI [−0.65, 0.37]). See Fig. S1 for a graphical summary of the results.

### Aggression has no effect on morphology across individuals and a negligible effect at the population level (Q5)

Across all analyses, facial shape showed no consistent association with individual-level self-reported aggression. In the within–between models, the within-population component of self-reported aggression (W.TAGR) did not significantly predict facial shape in either women or men, and this applied to both enface and profile views (all *P* ≥ 0.21; *R*^2^ ≤ 0.002). This null effect persisted when age and BMI were included either as global covariates or as within-population centered covariates. This indicates that the absence of within-individual effects was robust to alternative control strategies.

In contrast, the between-population component of self-reported aggression (B.TAGR) was consistently associated with facial shape across sexes and facial views (all *P* = 0.001; *R*^2^ = 0.018–0.039), although these effects are small to medium. Age (*R*^2^ = 0.022–0.031) and BMI (*R*^2^ = 0.016–0.031) also exhibited significant associations with facial shape across all models. Interaction models have further revealed significant TAGR × population terms, which indicates heterogeneity in population-level associations. Nevertheless, these effects did not translate into detectable within-population relationships. The between-population effect reflects the fact that populations differ in their mean facial shape and these mean shape differences are not arbitrary: they align with population-level variation in aggression. On the other hand, once population-specific effects were partialized out, B.TAGR accounted for an extremely small proportion of the overall facial shape variation (*R*^2^ = 0.002). See Table S9 for the numerical summary of within- and between-population effects of aggression on facial shape.

## Discussion

Our investigation of 16 culturally, geographically, and biologically diverse populations showed that facial sexual dimorphism is not a reliable marker of self-reported aggression at the individual level. Furthermore, population-level variation in self-reported aggression does not covary with facial SShD and overall masculinization. These null results are robust across frontal and profile facial configurations, the sexes, and across complementary analytical frameworks. Across analyses, the estimated effects were consistently small and centered near zero, with compatibility intervals providing little indication of meaningful associations.

Our findings align closely with growing skepticism regarding any consistent link between facial appearance and aggression. In fact, they suggest that inconsistent prior results were driven by fundamental methodological challenges inherent to reductive metrics such as the fWHR (47): as a ratio variable, fWHR is mathematically vulnerable to artifacts which produce spurious correlations (48). Moreover, fWHR is heavily confounded by facial allometry and BMI, which makes it nonindependent of body size (16, 49–51). Critically, when width and height are modeled as independent predictors, the purported fWHR effects often disappear (52, 53).

This methodological fragility elucidates why standardized anthropometry (15) and large-scale reanalyses of over 130,000 individuals (7) have failed to detect any robust associations between facial structure and aggression-related traits. Furthermore, the directionality of the metric is often inconsistent with the “aggressive face” hypothesis. For instance, males from the general Mexican population exhibit on average a higher fWHR than homicide offenders (18), and recent cross-cultural work in Nigeria and Indonesia found only weak or nonexistent correlations with aggression, independent of sex (14, 54). Our results demonstrate that even comprehensive geometric morphometric representations reveal no systematic links to aggression. It seems therefore that, in an era of high-dimensional morphometrics and AI-powered automated phenotyping, reliance on reductive metrics such as fWHR obscures the complex realities of facial morphology and should be abandoned.

Sexually dimorphic facial morphology has often been discussed in the context of androgen-related development and intrasexual competition, which led to the expectation that more masculinized faces may be associated with greater aggressiveness (31, 55). This expectation aligns with evidence that facial masculinity and fWHR increase perceived aggression and dominance (56). It is also consistent with models which suggest that dominance is one of the main dimensions of social evaluations of faces (3, 4), a structure that also recovers across world regions when Todorov's analytical approach is applied (57). The magnitude of these perceptual effects, however, depends heavily on study design. Dong et al. (58) showed that the large effects (Cohen's d's of 2.51 and 3.28) of face-shape masculinity on perceived dominance obtained with two-alternative forced-choice tasks and computer-manipulated stimuli become considerably smaller (Cohen's d 0.44 and 0.62), and in two of the three studies nonsignificant, when unmanipulated faces are rated and masculinity is measured morphometrically—that is, under conditions closest to those employed here. Even the perceptual side of the masculinity-dominance link is therefore weaker than literature based on manipulated stimuli implies. Furthermore, perceptual consensus does not imply behavioral validity. The functional basis of face evaluation suggests that trait inferences arise from an overgeneralization of cues linked to threat detection (3), and the error management theory predicts a bias toward overattributing potential threat under uncertainty (59, 60). Empirical work on threat-based stereotyping further demonstrates that facial features associated with danger can systematically shape impressions quite independently of actual behavioral dispositions (61, 62). In this context, the absence of association between facial masculinity and self-reported aggression in our data suggests that morphologically masculinized facial structure is not a reliable marker of aggressiveness or dispositional aggression. Rather than reflecting stable aggressive tendencies, widely shared inferences from facial masculinity may reflect generalized perceptual heuristics which are but weakly, if at all, calibrated to self-reported personality traits.

This decoupling extends to the between-population level. Contrary to expectations (Q2–Q4), populations with higher mean aggression exhibit neither a more pronounced SShD (Q2), nor a more masculinized average phenotype (Q3), nor wider sex differences in behavioral tendencies (Q4). This result should, however, be interpreted with the appropriate level of analysis in mind. The well-documented association between sexual dimorphism and male–male competition in nonhuman primates is an interspecific pattern: across species, dimorphism in body mass and canine size scales with surrogate measures of the intensity of intrasexual selection, such as mating system and operational sex ratio (30). This macroevolutionary signal does not translate into the prediction that, within a single species, populations with higher aggregate aggression should be more dimorphic, and, to our knowledge, no primate study has tested such associations at the population level. Human facial shape dimorphism was most likely shaped by the same broad contest-competition pressures as those acting in other primates; crucially, however, the resulting dimorphism also reflects a deep evolutionary history, including climatic and subsistence adaptations and possibly genetic drift (36, 40, 63–66), rather than plastically tracking contemporary population-level differences in aggression.

The substantial between-population variation in baseline aggression probably reflects cross-cultural differences in the expression, regulation, and reporting of aggressive tendencies, as well as potential measurement noninvariance, rather than straightforward differences in underlying biologically canalized behavioral propensities (67). This dissociation was further reinforced by multivariate shape regressions (Q5), which showed that self-reported aggression does not map onto the broader facial shape space. Even when analyzing holistic facial morphology, we found no specific “aggressive phenotypes.” While genetic substrates for aggression, such as WFS1 polymorphisms (68) or specific Y-haplogroups (69), undoubtedly exist, our results indicate they are not “inscribed” in the face.

It is important to differentiate between dispositional aggression (self-report) and its translation into overt action (behavior). These domains are related, but only rather loosely; the correlation is not strong and, moreover, it is often contextually conditioned (17, 70–72). Moreover, their overlap with androgen-dependent facial masculinization seems limited (5, 7, 13, 15, 73). Consequently, the hypothesis that faces signal aggressive intent requires a precise triangulation between self-reported affect, behavioral enactment, and morphology—a synergy that appears statistically elusive. Our findings suggest that the human face functions not as a transparent window into stable personality traits but rather as an interactive “social mask.” Insofar as the face serves to manage the impression one makes, however, this is likely to involve its dynamic and soft-tissue aspects rather than the skeletal shape differences examined here; just as perceived trustworthiness does not reliably predict honesty, facial masculinization does not predict aggression.

It is therefore useful to distinguish among sexually dimorphic facial traits by their likely function. Some, such as facial hair, are plausibly communicative (74). The craniofacial shape differences examined here—robusticity of the mandible, the supraorbital region, and the lower face—may owe less to communication than to structural integrity. As Carrier and Morgan (75); see also Refs. (31, 76) have argued, the skeletal regions most frequently fractured in interpersonal violence are also the most robust and the most sexually dimorphic parts of the human skull. Mandibular fractures are several times more frequent in men than in women, and interpersonal assault ranks among the leading causes across clinical series, alongside the evolutionarily novel contribution of motor vehicle accidents. On this view, and as in other primates, the buttressing of the jaw and periorbital bones is more plausibly an adaptation for withstanding blows during male contests than a device for advertising threat potential.

This structural interpretation departs from accounts that treat the signaling of threat or dominance as the primary function of masculine facial shape (2, 77), and it fits our results. If masculine facial robusticity evolved mainly as protection during physical contests, it need not track aggressive personality, and our data indicate that it does not. In sum, while human faces convey a wide array of social information, aggression is not reliably written into facial SShD, either within or across populations.

One limitation of the present study concerns our reliance on self-reported aggression. Self-report is a genuinely informative approach here: HOST and ANGER are internal, affective dispositions, and self-report offers direct access to these dispositions in a way that complements what behavioral or observer ratings can capture (see the self–other knowledge asymmetry model; 78). Importantly, the BPAQ shows meaningful correlations with act-based aggression measures in Western samples (*r* = 0.60 physical, *r* = 0.52 verbal; 9), although this convergent validity has not been directly tested in the present populations, and internal consistency was acceptable throughout (*α* = 0.78–0.89; Table S4). It is also worth noting that observer-rated aggression carries its own source of bias, since judgments of aggressiveness are themselves shaped by facial masculinity (4), a confound self-report does not share. Self-reports nonetheless remain open to socially desirable responding, a concern potentially amplified where illiterate participants had items read aloud by an assistant; reassuringly, administration mode showed no systematic effect on scores (Section S5). Translation equivalence was checked by native speakers, and measurement invariance across the sixteen samples was not tested; between-population score differences should therefore be read with some caution. This bears chiefly on Q2–Q4, and less on the within-population analyses, where group-mean centering removes between-population differences in response style. Replication with behavioral or peer-report measures would nonetheless be valuable, particularly for the between-population comparisons.

A second limitation is that most of the sampled populations were from Asia. On the other hand, these sixteen subsamples are highly diverse, spanning a wide range of genetic backgrounds, ecological settings, and cultural practices—from the high-altitude Tibetan and Ladakhi groups to the coastal Sinhalese, from the historically isolated Kalash to large North Indian populations. Inclusion of the Cabo Verdean population with its unique combination of African and European genetic and cultural influences further enhances the diversity of our dataset and contributes to the meaningfulness of cross-population comparisons. In conjunction with evidence from other studies, we believe that our results provide a solid and well-argued answer to the question of a possible association between aggression and facial shape. Despite the abovementioned geographical limitation, our dataset represents an unprecedented collection of ethnic groups that were never jointly studied in biopsychological and cross-cultural research. It should moreover be noted that our study is based on data drawn from facial photographs and questionnaires collected by a single team under standardized conditions (as opposed to a meta-analytic compilation based on the work of multiple independent research groups).

## Conclusions

Collectively, our results challenge the long-standing assumption that facial sexual dimorphism functions as an honest signal of aggressive disposition. We found no consistent covariation between facial morphology and self-reported aggression either at individual level, or across populations. Facial masculinization and aggression varied independently and cross-population differences in aggression did not follow changes in morphological dimorphism. These findings undermine models which work with the notion of a shared androgenic or sexual-selection-driven architecture that links craniofacial development with aggressive personality. Rather than serving as a biologically transparent cue of aggression, it seems that the human face is decoupled from dispositional aggressiveness. Any inference of aggressive character from facial morphology is more consistent with perceptual heuristics than with biologically deterministic interpretation. We would emphasize that these null findings are the substantive result rather than the absence of one. They rest on high-dimensional shape data rather than a single ratio, on 16 populations rather than one, and on models that accommodate both confounds and cross-population heterogeneity; and they hold consistently across sexes, facial views, and levels of analysis. A natural extension of this design would be to apply the same morphometric and multipopulation approach to perceived aggressiveness and dominance, which would establish whether the perceptual consensus reported for manipulated stimuli holds for unmanipulated faces across cultures.

## Supplementary Material

pgag299_Supplementary_Data
