# Supplementary material for: Self-reported aggressiveness does not follow facial masculinization in diverse South Asian populations
Source: PNAS Nexus. 2026 Sep 4;5(9):pgag299. doi: 10.1093/pnasnexus/pgag299 (PMC13596738; doi:10.1093/pnasnexus/pgag299)
Supplement: Supplementary file 1 [file pgag299_supplementary_data.docx]

**SUPPLEMENTARY MATERIALS**

**Self-reported aggressiveness does not follow facial masculinization in diverse South Asian populations**

Karel Kleisner*^1^, Zuzana Štěrbová^1,2^, Vojtěch Fiala^1,11^, Eliška Podgorná^3^, Abhishikta Ghosh Roy^4^, Banita Behera^4^, Deepika Rani^5^, Kewal Krishan^5^, Stanzin Namga^6^, Sanzida Khatun^7^, Abdul Raheem Jesmil^8^, Abdul Hameed^9^, Viktor Černý^3,10^

^1^ Department of Philosophy and History of Science, Faculty of Science, Charles University, Prague, Czech Republic

^2^ Department of Psychology, Faculty of Arts, Charles University, Prague, Czech Republic

^3^ Department of Natural Sciences and Archaeometry, Institute of Archaeology, Czech Academy of Sciences, Prague, Czech Republic

^4^ Anthropological Survey of India, Ministry of Culture, Government of India, India

^5^ Department of Anthropology, Panjab University, Chandigarh, India

^6^ Department of Geology, University of Jammu, Jammu, UT of Jammu and Kashmir, India

^7^ Department of Anatomy, Birat Medical College Teaching Hospital, Morang, Nepal

^8^ Abdul Raheem Jesmil, Department of Archaeology, Ministry of Buddhasasana, Religious and Cultural Affairs, Sri Lanka

^9^ Department of Archaeology, Hazara University Mansehra, Pakistan

^10^ Department of Anthropology and Human Genetics, Charles University, Prague, Czech Republic

^11^ Institute of Advanced Studies, Nicolaus Copernicus University in Toruń, Poland.

*Correspondence: [karel.kleisner@natur.cuni.cz](mailto:karel.kleisner@natur.cuni.cz)

**Table S1**. Population characteristics, questionnaire administration, and linguistic background

| **Population Code** | **Population Name** | **Literacy** | **Format** | **Completion Type** | **Longitude** | **Latitude** | **Language family** |
| --- | --- | --- | --- | --- | --- | --- | --- |
| KON | Kondh | 0% | PaP | 100% Assisted | 82.73 | 18.86 | Dravidian |
| GAD | Gadaba | 0% | PaP | 100% Assisted | 82.73 | 18.86 | Austroasiatic |
| THA | Tharu | 25% | PaP | 75% Assisted | 87.28 | 26.46 | Indo-Aryan |
| CTR | Chhetri | 0% | PaP | 100% Assisted | 85.83 | 27.59 | Indo-Aryan |
| SRP | Sherpa | 50% | PaP | 50% Assisted | 86.78 | 27.56 | Tibeto-Burman |
| LAD | Ladakhi | 90% | PaP | 10% Assisted | 77.58 | 34.15 | Tibeto-Burman |
| SIND | Sindhi | 90% | PaP | 10% Assisted | 66.98 | 24.86 | Indo-Aryan |
| CVST | Cape Verdeans | 100% | PaP | Self | -23.51 | 14.92 | Indo-European |
| SIN | Sinhalese | 90% | PaP | 10% Assisted | 80.73 | 6.20 | Indo-Aryan |
| TIB | Tibetans | 50% | PaP | 50% Assisted | 77.58 | 34.15 | Tibeto-Burman |
| BEN | Bengali | 90% | PaP | 10% Assisted | 90.62 | 23.87 | Indo-Aryan |
| KAL | Kalash | 0% | PaP | 100% Assisted | 71.69 | 35.70 | Indo-Aryan |
| TA | Tamang | 25% | PaP | 75% Assisted | 85.83 | 27.59 | Tibeto-Burman |
| KAS | Kashmiri | 90% | PaP | 10% Assisted | 73.48 | 34.36 | Indo-Aryan |
| TAM | Tamil | 90% | PaP | 10% Assisted | 80.11 | 9.79 | Dravidian |
| IND | India | 90% | PaP | 10% Assisted | 76.70 | 31.05 | Indo-Aryan |

**Note.** **Literacy** indicates the estimated percentage of participants within each population who were able to read and complete the questionnaire independently. **Format** refers to the medium used to complete the questionnaire: *Online* (on a computer or mobile device) or *Paper-and-pencil (PaP)*. **Completion Type** reflects the estimated percentage of participants in each population who completed the questionnaire with assistance (*Assisted*); the remaining participants completed it independently (Self). In assisted cases, a research assistant read the items aloud and recorded responses. **Data Collection Context** provides a brief description of the data collection setting and circumstances, including any procedures to ensure privacy, participant instructions, and incentives. **Language family** indicates the linguistic family of the language into which the questionnaire was translated and in which participants completed the questionnaire.

**Section S1.** Questionnaire administration procedures across populations

India – questionnaires were translated into Hindi language and administered individually or in groups. The population of northern India was sampled in the state of Himachal Pradesh in Government Model Industrial Training Institute (ITI) Nalagarh and the town of Nalagarh. In the case of a group of students, the questions were read aloud with additional explanations in Hindi and the participants then filled in the individual answers independently. Other Indian populations were sampled in the Ladakh – these were Ladakhi and Tibetans. The questionnaires were translated into Ladakhi and Tibetan languages. Here, data was collected at The University of Ladakh among students and in some public spaces in the city of Leh. The tribal populations of Gadaba and Khond were sampled in several villages around the city of Koraput in the state of Odisha. Both populations speak their own dialect (Gutab and Kui respectively), which does not have a written form. The questionnaires were therefore filled in by assistants who interviewed the respondents in the Odia language, which both groups understand.

Nepal – questionnaires were translated into Nepali, the official language of Nepal understood by all its inhabitants. The Tharu were contacted in the city of Biratnagar and its vicinity. The research was conducted in public spaces, in public buildings or in the courtyard of local assistants. In most cases, the questions were read and the respondents answered them either individually or in groups. Data were obtained in a similar way from the Tamang and Chhetri in the village of Majipheda, about 60 km east of Kathmandu. The Sherpa were sampled in the village of Juke, located in northern Nepal near Sagarmatha National Park.

Pakistan – questionnaires were translated into Urdu, the national language of Pakistan. The Sindhi were sampled in University of Karachi, where the questionnaires were filled out by students and teachers individually. However, due to the smaller number of women, we contacted part of the Sindhi population in public spaces in the city outskirts. The Kashmiri population was sampled in the city of Muzaffarabad and its immediate surroundings, including the University of Azad Jammu & Kashmir. Most respondents completed the questionnaires independently. The Kalash population was sampled in the village of Bumburait, one of the few villages in the Chitral region in the northwest of the country where these people still live. The questionnaires were read and the answers recorded by an administrator.

Bangladesh – for the Bengalis population, the questionnaires were translated into Bengali language and were mostly completed by the people themselves. The data were obtained from the village population in the Bonaid locality approximately 50 km north of Dhaka capital.

Sri Lanka – for the Tamil population, the questionnaires were translated into Tamil language, and the sampling took place in the north of the island in the city of Jaffna and its immediate surroundings. The respondents were able to complete the questionnaires independently in most cases. For the Sinhalese population, we translated the questionnaires into Sinhala language, and we contacted the population in the city of Muruthawela in the south of the island approximately 50 km northeast of Matara. Here too, respondents were able to complete the questionnaires in most cases independently.

Cape Verde – the questionnaires were translated into Portuguese Creole. The Cape Verdean population was contacted on Santiago Island, in the city of Praia, at the University of Cape Verde and in the country's public administration offices. All participants completed the questionnaires independently.

**Section S2.** Questionnaire Description and Translation Procedures

We administered the Buss–Perry Aggression Questionnaire (AQ; Buss & Perry, 1992), a 29-item self-report measure widely used cross-culturally to assess dispositional aggression. The AQ consists of four subscales: Physical Aggression (9 items), Verbal Aggression (5 items), Anger (7 items), and Hostility (8 items). With the exception of the Kashmiri and Kalas samples, all participants responded on a 1–5 scale. Kashmiri and Kalas participants used a 1–7 response format; these responses were linearly rescaled to the 1–5 metric using the transformation: new = ((old − 1) × 4 / 6) + 1.

All questionnaires were translated into the respective local languages by native speakers using a single forward-translation procedure. No back-translation was conducted; instead, translations were checked for clarity and conceptual equivalence by additional native speakers familiar with psychological research. All procedures are summarized in Table S1.

**Section S3.** Descriptive Statistics of the Buss-Perry Agression Questionnaire

For each cultural group and each AQ subscale, we computed descriptive statistics including mean, variance, minimum, maximum, skewness, and kurtosis. These values help identify differences in response style across cultures, such as preferences for extreme vs. moderate responses or distributional irregularities. Strong skewness or kurtosis may reflect culturally patterned responding rather than substantive differences in aggression. All descriptive values are reported in Table S2.

**Table S2.** Descriptive statistics of the Buss–Perry Aggression Questionnaire by population

| **Population** | **Subscale** | **Mean** | **Variance** | **Min** | **Max** | **Skewness** | **Kurtosis** |
| --- | --- | --- | --- | --- | --- | --- | --- |
| KON | Total | 62.36 | 239.9401 | 29 | 141 | 1.44 | 10.01 |
|  | Physical Aggression | 20.37 | 20.7025 | 13 | 41 | 1.07 | 6.84 |
|  | Verbal Aggression | 11.12 | 11.4921 | 5 | 25 | 1 | 6.06 |
|  | Anger | 16.72 | 12.25 | 11 | 31 | 0.87 | 5.26 |
|  | Hostility | 17.36 | 27.1441 | 8 | 36 | 0.31 | 3.84 |
| GAD | Total | 56.64 | 138.2976 | 35 | 104 | 0.94 | 5.22 |
|  | Physical Aggression | 18.93 | 9.8596 | 13 | 33 | 1.11 | 7.01 |
|  | Verbal Aggression | 9.75 | 6.3001 | 5 | 18 | 0.45 | 3.42 |
|  | Anger | 15.64 | 7.1824 | 12 | 22 | 0.32 | 2.3 |
|  | Hostility | 16.29 | 14.7456 | 8 | 25 | 0.22 | 2.4 |
| THA | Total | 63.03 | 222.6064 | 31 | 106 | 0.60 | 2.86 |
|  | Physical Aggression | 20.52 | 23.7169 | 13 | 33 | 0.21 | 2.38 |
|  | Verbal Aggression | 14.03 | 13.8384 | 5 | 22 | -0.03 | 2.76 |
|  | Anger | 13.58 | 17.7241 | 7 | 25 | 0.54 | 2.63 |
|  | Hostility | 15.20 | 54.6121 | 8 | 32 | 0.68 | 2.18 |
| CTR | Total | 61.83 | 212.5764 | 37 | 118 | 1.15 | 4.96 |
|  | Physical Aggression | 20.14 | 15.0544 | 13 | 32 | 0.87 | 3.31 |
|  | Verbal Aggression | 10.75 | 8.5264 | 5 | 21 | 1.10 | 4.47 |
|  | Anger | 17.39 | 13.6161 | 11 | 28 | 0.90 | 3.25 |
|  | Hostility | 17.04 | 23.1361 | 8 | 34 | 1.22 | 5.32 |
| SRP | Total | 65.88 | 296.1841 | 29 | 108 | -0.33 | 3.11 |
|  | Physical Aggression | 20.45 | 23.0400 | 13 | 35 | 0.64 | 3.42 |
|  | Verbal Aggression | 11.73 | 13.8384 | 5 | 22 | -0.23 | 2.70 |
|  | Anger | 16.06 | 18.3184 | 7 | 26 | 0.17 | 2.53 |
|  | Hostility | 18.50 | 32.1489 | 8 | 32 | -0.19 | 2.54 |
| LAD | Total | 72.81 | 178.4896 | 42 | 98 | -0.64 | 2.81 |
|  | Physical Aggression | 22.82 | 23.7169 | 10 | 33 | -0.28 | 2.80 |
|  | Verbal Aggression | 12.93 | 12.0409 | 7 | 24 | 1.03 | 4.42 |
|  | Anger | 18.44 | 10.7584 | 10 | 26 | -0.46 | 3.04 |
|  | Hostility | 20.58 | 23.9121 | 7 | 30 | -0.67 | 3.48 |
| SIND | Total | 75.78 | 222.01 | 48 | 114 | 0.55 | 2.72 |
|  | Physical Aggression | 24.01 | 21.2521 | 16 | 36 | 0.83 | 3.06 |
|  | Verbal Aggression | 14.68 | 14.7456 | 6 | 25 | 0.48 | 2.93 |
|  | Anger | 18.74 | 16.5649 | 11 | 31 | 0.77 | 3.54 |
|  | Hostility | 20.21 | 24.2064 | 11 | 35 | 0.69 | 3.54 |
| CVST | Total | 71.48 | 229.5225 | 42 | 118 | 0.70 | 3.89 |
|  | Physical Aggression | 19.89 | 30.8025 | 9 | 38 | 0.81 | 4.25 |
|  | Verbal Aggression | 13.68 | 11.1556 | 7 | 23 | -0.01 | 2.72 |
|  | Anger | 15.67 | 28.9444 | 7 | 30 | 0.57 | 2.93 |
|  | Hostility | 20.76 | 39.3129 | 8 | 38 | 0.39 | 2.90 |
| SIN | Total | 68.63 | 303.4564 | 29 | 116 | -0.18 | 2.55 |
|  | Physical Aggression | 19.99 | 29.0521 | 9 | 34 | 0.28 | 3.01 |
|  | Verbal Aggression | 13.80 | 24.9001 | 5 | 22 | -0.02 | 1.98 |
|  | Anger | 17.27 | 22.4676 | 7 | 27 | -0.13 | 2.53 |
|  | Hostility | 18.34 | 38.4400 | 8 | 33 | 0.28 | 2.60 |
| TIB | Total | 77.24 | 287.6416 | 41 | 129 | 0.04 | 3.38 |
|  | Physical Aggression | 22.83 | 36.3609 | 13 | 41 | 0.36 | 3.26 |
|  | Verbal Aggression | 13.56 | 15.6025 | 5 | 24 | -0.19 | 2.88 |
|  | Anger | 19.34 | 19.0969 | 7 | 28 | -0.48 | 3.06 |
|  | Hostility | 21.71 | 29.5936 | 10 | 32 | -0.13 | 2.37 |
| BEN | Total | 80.90 | 602.7025 | 29 | 145 | 0.92 | 3.75 |
|  | Physical Aggression | 22.52 | 60.3729 | 10 | 43 | 1.01 | 3.55 |
|  | Verbal Aggression | 14.42 | 28.9444 | 5 | 25 | 0.32 | 2.62 |
|  | Anger | 20.19 | 24.3049 | 10 | 31 | 0.29 | 2.97 |
|  | Hostility | 23.87 | 47.3344 | 8 | 40 | 0.70 | 3.3 |
| KAL | Total | 82.09 | 267.6496 | 36 | 123 | 0.26 | 3.05 |
|  | Physical Aggression | 24.28 | 26.1121 | 17 | 42 | 0.92 | 3.65 |
|  | Verbal Aggression | 14.54 | 11.4921 | 5 | 25 | 0.09 | 3.84 |
|  | Anger | 18.78 | 24.1081 | 12 | 30 | 0.56 | 2.26 |
|  | Hostility | 24.32 | 26.3169 | 9 | 33 | -0.45 | 3.19 |
| TA | Total | 80.49 | 245.5489 | 36 | 134 | 0.00 | 4.50 |
|  | Physical Aggression | 23.85 | 17.2225 | 16 | 38 | 0.62 | 4.12 |
|  | Verbal Aggression | 14.10 | 10.5625 | 5 | 21 | -0.29 | 3.13 |
|  | Anger | 20.56 | 9.7969 | 11 | 31 | 0.10 | 4.30 |
|  | Hostility | 23.15 | 27.7729 | 12 | 37 | 0.07 | 2.92 |
| KAS | Total | 86.02 | 268.3044 | 38 | 130 | 0.11 | 3.69 |
|  | Physical Aggression | 24.94 | 32.7184 | 13 | 38 | 0.27 | 2.56 |
|  | Verbal Aggression | 15.65 | 15.4449 | 7 | 25 | 0.50 | 2.76 |
|  | Anger | 19.96 | 27.2484 | 8 | 31 | 0.28 | 2.51 |
|  | Hostility | 25.07 | 29.5936 | 11 | 40 | 0.25 | 3.43 |
| TAM | Total | 87.99 | 316.4841 | 34 | 128 | -0.52 | 3.15 |
|  | Physical Aggression | 20.53 | 41.3449 | 9 | 36 | 0.37 | 2.34 |
|  | Verbal Aggression | 16.96 | 20.7936 | 5 | 25 | -0.41 | 2.72 |
|  | Anger | 22.58 | 27.2484 | 11 | 35 | -0.2 | 2.61 |
|  | Hostility | 27.29 | 44.7561 | 10 | 40 | -0.48 | 2.48 |
| IND | Total | 91.80 | 227.4064 | 33 | 134 | -0.67 | 5.26 |
|  | Physical Aggression | 26.86 | 29.9209 | 13 | 39 | -0.32 | 3.10 |
|  | Verbal Aggression | 16.61 | 12.6025 | 5 | 24 | -0.33 | 2.96 |
|  | Anger | 19.92 | 20.4304 | 7 | 31 | -0.02 | 3.60 |
|  | Hostility | 28.70 | 24.6016 | 8 | 39 | -0.88 | 4.69 |

**Note.** Total Score range: 29–145; Physical Aggression: 9–45; Verbal Aggression: 5–25; Anger: 7–35; Hostility: 8–40.

**Section S4.** Handling Missing Data

Missing values were evaluated at the level of each subscale. When fewer than 50% of items were missing, missing values were replaced by the respondent’s person-mean for that subscale (Graham et al., 2003). When 50% or more items were missing, that subscale score was coded as missing and excluded from subsequent analyses. The number of initially considered cases, as well as the final size of the dataset, is summarized in Table S3.

**Table S3.** Initial sample sizes, exclusions, and final analytic sample across populations.

| **Population** | Sex | N_Oring_Prof | N_Oring_Enf | Final_N | Sex | N_Oring_Prof | N_Oring_Enf | Final_N |
| --- | --- | --- | --- | --- | --- | --- | --- | --- |
| KON | F | 51 | 51 | 39 | M | 66 | 66 | 42 |
| GAD | F | 50 | 49 | 29 | M | 50 | 50 | 46 |
| THA | F | 50 | 50 | 50 | M | 55 | 55 | 52 |
| CTR | F | 53 | 53 | 51 | M | 52 | 51 | 48 |
| SRP | F | 60 | 56 | 56 | M | 59 | 59 | 57 |
| LAD | F | 57 | 57 | 31 | M | 43 | 43 | 26 |
| SIND | F | 51 | 51 | 49 | M | 55 | 55 | 52 |
| CVST | F | 55 | 55 | 53 | M | 54 | 54 | 52 |
| SIN | F | 59 | 60 | 51 | M | 52 | 52 | 55 |
| TIB | F | 47 | 47 | 43 | M | 34 | 34 | 27 |
| BEN | F | 50 | 50 | 48 | M | 52 | 52 | 52 |
| KAL | F | 56 | 56 | 50 | M | 46 | 46 | 45 |
| TA | F | 51 | 51 | 37 | M | 46 | 46 | 35 |
| KAS | F | 45 | 45 | 40 | M | 50 | 50 | 44 |
| TAM | F | 56 | 56 | 53 | M | 44 | 44 | 43 |
| IND | F | 62 | 62 | 62 | M | 60 | 60 | 60 |
| **Summary** | **F** | **853** | **849** | **742** | **M** | **818** | **817** | **736** |
| **Note.** Variables from left to right: culture and sex are idiosyncratic, N_Orig_Prof, N_Orig_Enf correspond to all available specimens for profile and enface photos while Final N is after subsetting. The row entitled "Summary" is for all the stimuli | | | | | | | | |

**Section S5.** Reliability Computation and Interpretation

Cronbach’s alpha was computed in SPSS Version 31 using the Reliability procedure (Model: Alpha), excluding both user-missing and system-missing values. Alpha was calculated separately for each cultural group, for the total scale, and each of the four subscales. This approach acknowledges potential cross-cultural differences in response patterns and item interpretation and avoids misleading pooled estimates.

Populations with total-scale alpha ≥ .70 were retained for main analyses, following psychometric recommendations that values at or above .70 indicate acceptable internal consistency for research purposes (Tavakol & Dennick, 2011). Reliability estimates from the original validation study (Buss & Perry, 1992) are presented alongside our estimates in Table S4 for comparison. Across the majority of populations, total-scale reliability was comparable to or slightly lower than the original values, which is common in cross-cultural field research with heterogeneous samples.

Lower reliability values in some groups (particularly for certain subscales) may reflect small sample sizes, limited literacy, or cultural differences in item comprehension. In several field settings, questionnaires were administered orally to illiterate participants, which may have reduced the precision and consistency of responses. In addition, some constructs may not map cleanly onto local cultural categories of emotion or behavior, which can further reduce internal consistency. All alpha values (non-standardized) are reported in Table S4. Nevertheless, the supplementary analysis in Section S5 below shows that administration procedure did not systematically affect the total aggression scores.

**Table S4:** Cronbach’s alpha for total scale and subscales across populations

| **Population** | **Total** | **Physical Agression** | **Verbal Agression** | **Anger** | **Hostility** | **Notes** |
| --- | --- | --- | --- | --- | --- | --- |
| **Original paper** | .89 | .85 | .72 | .83 | .77 | Buss & Perry (1992) |
| KON | .867 | .680 | .656 | .517 | .827 |  |
| GAD | .832 | .481 | .673 | .390 | .770 |  |
| THA | .823 | .559 | .685 | .540 | .893 |  |
| CTR | .890 | .673 | .702 | .613 | .820 |  |
| SRP | .826 | .496 | .535 | .405 | .683 |  |
| LAD | .810 | .617 | .631 | .412 | .777 |  |
| SIND | .857 | .591 | .722 | .587 | .751 |  |
| CVST | .880 | .735 | .648 | .210 | .812 |  |
| SIN | .806 | .612 | .628 | .459 | .733 |  |
| TIB | .846 | .668 | .657 | .541 | .697 |  |
| BEN | .881 | .737 | .708 | .402 | .689 |  |
| KAL | .838 | .547 | .477 | .648 | .679 | Used 1–7 scale; rescaled |
| TA | .868 | .569 | .667 | .322 | .761 |  |
| KAS | .828 | .605 | .567 | .621 | .635 | Used 1–7 scale; rescaled |
| TAM | .781 | .572 | .537 | .455 | .709 |  |
| IND | .795 | .566 | .375 | .441 | .585 |  |
| BRH | .438 | .733 | −.082 | −.960 | .492 | Excluded |

**Table S5:** Descriptive statistics of the participants by population

| **Population** | **N** | **Sex** | **Age (M ± SD [range]) [NAs]** | **Weight (M ± SD [range]) [NAs]** | **Height (M ± SD [range]) [NAs]** | **BMI (M ± SD [range]) [NAs]** |
| --- | --- | --- | --- | --- | --- | --- |
| KON | 81 | MF | 35.91 ± 14.89 (18.00–69.00) [0] | 50.66 ± 7.73 (40.00–73.00) [10] | 155.06 ± 7.37 (141.00–169.00) [0] | 20.73 ± 2.29 (16.73–27.82) [10] |
| GAD | 75 | MF | 40.26 ± 14.42 (18.00–69.00) [1] | 51.50 ± 5.92 (40.00–67.00) [9] | 158.40 ± 8.29 (137.00–176.00) [0] | 20.19 ± 2.05 (16.41–25.53) [9] |
| THA | 102 | MF | 39.65 ± 16.23 (17.00–77.00) [3] | 59.11 ± 9.69 (40.00–90.00) [14] | 158.81 ± 10.43 (134.00–183.00) [17] | 23.54 ± 3.78 (14.69–31.22) [20] |
| CTR | 99 | MF | 45.51 ± 14.24 (18.00–70.00) [0] | 54.64 ± 10.88 (40.00–91.00) [9] | 153.92 ± 8.71 (135.00–172.00) [0] | 22.79 ± 3.94 (17.09–34.25) [9] |
| SRP | 113 | MF | 43.75 ± 15.13 (17.00–91.00) [0] | 62.49 ± 9.42 (42.00–95.00) [0] | 158.85 ± 7.10 (145.00–182.00) [0] | 24.77 ± 3.53 (17.72–36.65) [0] |
| LAD | 57 | MF | 35.63 ± 14.23 (22.00–80.00) [0] | 59.69 ± 8.98 (40.00–85.00) [2] | 159.13 ± 7.38 (142.00–175.00) [5] | 23.81 ± 3.37 (18.69–36.79) [6] |
| SIND | 101 | MF | 30.02 ± 12.23 (19.00–72.00) [1] | 59.46 ± 12.72 (40.00–108.00) [6] | 163.10 ± 10.44 (134.00–189.00) [0] | 22.06 ± 4.10 (15.32–36.51) [6] |
| CVST | 105 | MF | 39.37 ± 12.72 (19.00–62.00) [0] | 75.83 ± 13.70 (47.00–105.00) [0] | 170.80 ± 9.27 (150.00–191.00) [0] | 26.04 ± 4.60 (17.37–37.20) [0] |
| SIN | 106 | MF | 53.96 ± 13.43 (19.00–83.00) [2] | 59.39 ± 11.27 (41.00–106.00) [3] | 159.13 ± 8.82 (143.00–180.00) [3] | 23.38 ± 3.93 (15.06–36.25) [5] |
| TIB | 70 | MF | 38.70 ± 13.45 (18.00–78.00) [4] | 63.67 ± 11.92 (40.00–98.00) [3] | 161.11 ± 7.73 (137.00–175.00) [7] | 24.45 ± 3.99 (16.65–36.21) [8] |
| BEN | 100 | MF | 34.70 ± 13.36 (18.00–80.00) [2] | 54.98 ± 8.66 (40.00–85.00) [9] | 155.45 ± 8.29 (133.00–175.00) [1] | 22.57 ± 3.60 (16.56–37.28) [9] |
| KAL | 95 | MF | 32.17 ± 15.23 (15.00–80.00) [0] | 55.44 ± 11.33 (40.00–85.00) [2] | 155.04 ± 12.16 (130.00–185.00) [1] | 23.21 ± 3.94 (15.06–38.05) [3] |
| TA | 72 | MF | 41.58 ± 12.10 (18.00–60.00) [0] | 55.90 ± 8.51 (40.00–82.00) [3] | 157.08 ± 10.78 (130.00–183.00) [6] | 22.67 ± 3.59 (14.93–35.50) [7] |
| KAS | 84 | MF | 26.63 ± 9.94 (18.00–58.00) [1] | 59.65 ± 12.69 (40.00–93.00) [3] | 160.28 ± 12.08 (131.00–183.00) [5] | 23.35 ± 5.09 (14.69–35.71) [7] |
| TAM | 96 | MF | 39.13 ± 15.20 (17.00–72.00) [2] | 60.54 ± 11.61 (40.00–90.00) [4] | 160.66 ± 8.94 (145.00–183.00) [0] | 23.50 ± 4.07 (13.84–34.72) [4] |
| IND | 122 | MF | 26.76 ± 13.80 (17.00–72.00) [0] | 59.12 ± 13.80 (40.00–110.00) [4] | 162.57 ± 10.69 (140.00–200.00) [0] | 22.20 ± 4.31 (15.35–36.51) [4] |

**Note.** Values are presented as mean ± standard deviation, with observed range in parentheses and number of missing values in square brackets. MF = combined male and female sample. Height is reported in centimeters, weight in kilograms, and BMI in kg/m².

**Table S5 [continued]**
Descriptive statistics of SSHD indices by population and sex

| **Population** | **N** | **Sex** | **SSHD_enface_global (M ± SD [range] [NAs])** | **SSHD_profile_global (M ± SD [range] [NAs])** | **SSHD_enface_local (M ± SD [range] [NAs])** | **SSHD_profile_local (M ± SD [range] [NAs])** |
| --- | --- | --- | --- | --- | --- | --- |
| KON | 81 | MF | -0.0001 ± 0.0003 (-0.0009–0.0009) [0] | -0.0002 ± 0.0008 (-0.0020–0.0020) [0] | -0.0004 ± 0.0005 (-0.0019–0.0009) [0] | -0.0005 ± 0.0007 (-0.0023–0.0007) [0] |
| GAD | 75 | MF | 0.0000 ± 0.0004 (-0.0007–0.0008) [0] | -0.0002 ± 0.0009 (-0.0030–0.0021) [0] | 0.0000 ± 0.0005 (-0.0013–0.0012) [0] | 0.0000 ± 0.0014 (-0.0055–0.0021) [0] |
| THA | 102 | MF | 0.0002 ± 0.0003 (-0.0005–0.0010) [0] | 0.0001 ± 0.0008 (-0.0019–0.0020) [0] | 0.0004 ± 0.0007 (-0.0015–0.0017) [0] | 0.0003 ± 0.0010 (-0.0028–0.0029) [0] |
| CTR | 99 | MF | 0.0003 ± 0.0004 (-0.0005–0.0015) [0] | 0.0003 ± 0.0010 (-0.0018–0.0030) [0] | 0.0002 ± 0.0004 (-0.0007–0.0014) [0] | 0.0003 ± 0.0007 (-0.0012–0.0022) [0] |
| SRP | 113 | MF | -0.0002 ± 0.0003 (-0.0011–0.0006) [0] | -0.0006 ± 0.0010 (-0.0032–0.0018) [0] | -0.0001 ± 0.0003 (-0.0012–0.0008) [0] | -0.0006 ± 0.0019 (-0.0047–0.0040) [0] |
| LAD | 57 | MF | 0.0000 ± 0.0004 (-0.0008–0.0011) [0] | -0.0003 ± 0.0009 (-0.0018–0.0018) [0] | 0.0001 ± 0.0008 (-0.0015–0.0020) [0] | 0.0003 ± 0.0009 (-0.0027–0.0021) [0] |
| SIND | 101 | MF | -0.0001 ± 0.0004 (-0.0010–0.0010) [0] | 0.0004 ± 0.0010 (-0.0017–0.0023) [0] | 0.0002 ± 0.0011 (-0.0031–0.0023) [0] | 0.0003 ± 0.0013 (-0.0027–0.0028) [0] |
| CVST | 105 | MF | 0.0000 ± 0.0005 (-0.0013–0.0011) [0] | -0.0002 ± 0.0010 (-0.0025–0.0023) [0] | 0.0000 ± 0.0009 (-0.0026–0.0021) [0] | -0.0002 ± 0.0016 (-0.0039–0.0044) [0] |
| SIN | 106 | MF | 0.0000 ± 0.0003 (-0.0008–0.0009) [0] | 0.0003 ± 0.0009 (-0.0019–0.0025) [0] | 0.0002 ± 0.0007 (-0.0012–0.0022) [0] | 0.0001 ± 0.0006 (-0.0016–0.0014) [0] |
| TIB | 70 | MF | -0.0002 ± 0.0003 (-0.0009–0.0007) [0] | -0.0007 ± 0.0008 (-0.0025–0.0014) [0] | -0.0002 ± 0.0004 (-0.0010–0.0012) [0] | -0.0006 ± 0.0006 (-0.0018–0.0012) [0] |
| BEN | 100 | MF | 0.0001 ± 0.0004 (-0.0008–0.0015) [0] | 0.0002 ± 0.0011 (-0.0021–0.0027) [0] | 0.0000 ± 0.0004 (-0.0009–0.0008) [0] | 0.0002 ± 0.0020 (-0.0040–0.0046) [0] |
| KAL | 95 | MF | -0.0001 ± 0.0004 (-0.0010–0.0007) [0] | 0.0001 ± 0.0008 (-0.0018–0.0025) [0] | -0.0006 ± 0.0010 (-0.0026–0.0015) [0] | -0.0001 ± 0.0011 (-0.0042–0.0018) [0] |
| TA | 72 | MF | 0.0000 ± 0.0003 (-0.0006–0.0007) [0] | 0.0000 ± 0.0008 (-0.0021–0.0019) [0] | -0.0001 ± 0.0007 (-0.0020–0.0025) [0] | 0.0000 ± 0.0007 (-0.0016–0.0018) [0] |
| KAS | 84 | MF | 0.0000 ± 0.0005 (-0.0009–0.0010) [0] | 0.0004 ± 0.0010 (-0.0023–0.0026) [0] | 0.0000 ± 0.0014 (-0.0028–0.0029) [0] | 0.0008 ± 0.0019 (-0.0040–0.0060) [0] |
| TAM | 96 | MF | 0.0000 ± 0.0004 (-0.0009–0.0007) [0] | 0.0001 ± 0.0009 (-0.0023–0.0025) [0] | -0.0002 ± 0.0006 (-0.0016–0.0010) [0] | 0.0001 ± 0.0013 (-0.0028–0.0031) [0] |
| IND | 122 | MF | -0.0002 ± 0.0004 (-0.0010–0.0009) [0] | 0.0002 ± 0.0010 (-0.0022–0.0029) [0] | -0.0002 ± 0.0007 (-0.0018–0.0016) [0] | 0.0001 ± 0.0018 (-0.0036–0.0045) [0] |

**Note.** Values are presented as mean ± standard deviation (range) with the number of missing values in brackets. MF = combined male and female sample. SSHD = standardized sexual shape dimorphism index computed separately for enface and profile configurations at global and local levels.

**Figure S1. Between-population Bayesian analyses of self-reported aggression and facial sexual shape dimorphism.** Scatterplots show population-level relationships between total self-reported aggression (TAGR) and facial sexual shape dimorphism (SShD), analyzed separately for profile (top row) and enface (bottom row) facial configurations. Q2 tests whether populations with higher mean TAGR exhibit stronger facial sexual dimorphism (male–female difference in SShD). Q3 examines whether populations with more masculinized facial morphology, quantified as sex-adjusted mean SShD on the global axis, show higher mean TAGR. Q4 evaluates whether populations with greater sex differences in facial SShD also display greater sex differences in TAGR (male TAGR − female TAGR). Points represent population means, black lines indicate posterior mean regression slopes, and shaded areas represent 95% credible intervals from Bayesian linear regressions. Across all models, posterior estimates of the slope are small and their 95% credible intervals include zero, indicating no evidence for associations between population-level variation in self-reported aggression and facial dimorphism or masculinization in either profile or enface views.

**Table S6.** Summary of multilevel Bayesian model estimates for individual-level predictors of self-reported Total Aggression scores.

| **Multilevel Hyperparameters** | |  |  |  |  |  |  |
| --- | --- | --- | --- | --- | --- | --- | --- |
|  | **Estimate** | **Est.Error** | **l-95%CI** | **u-95%CI** | **Rhat** | **Bulk_ESS** | **Tail_ESS** |
| sd(intF) | 0.52 | 0.10 | 0.35 | 0.74 | 1.00 | 1031 | 1286 |
| sd(intM) | 0.53 | 0.10 | 0.37 | 0.76 | 1.00 | 1129 | 1381 |
| sd(profileF) | 0.06 | 0.05 | 0.00 | 0.18 | 1.00 | 1245 | 1251 |
| sd(profileM) | 0.07 | 0.06 | 0.00 | 0.22 | 1.00 | 1221 | 1446 |
| sd(enfaceF) | 0.07 | 0.06 | 0.00 | 0.22 | 1.00 | 1124 | 1191 |
| sd(enfaceM) | 0.07 | 0.05 | 0.00 | 0.19 | 1.00 | 1339 | 1169 |
| sd(bmiF) | 0.04 | 0.03 | 0.00 | 0.13 | 1.00 | 1584 | 1199 |
| sd(bmiM) | 0.07 | 0.05 | 0.00 | 0.18 | 1.00 | 974 | 822 |
| sd(ageF) | 0.12 | 0.07 | 0.01 | 0.29 | 1.00 | 829 | 1030 |
| sd(ageM) | 0.18 | 0.08 | 0.04 | 0.35 | 1.00 | 821 | 640 |
| cor(intF,intM) | 0.58 | 0.16 | 0.21 | 0.84 | 1.00 | 1313 | 1649 |
| cor(intF,profileF) | 0.11 | 0.28 | -0.47 | 0.63 | 1.00 | 3665 | 1453 |
| cor(intM,profileF) | 0.03 | 0.27 | -0.48 | 0.54 | 1.00 | 3578 | 1555 |
| cor(intF,profileM) | 0.03 | 0.27 | -0.51 | 0.55 | 1.00 | 3311 | 1208 |
| cor(intM,profileM) | -0.05 | 0.26 | -0.53 | 0.47 | 1.00 | 3373 | 1679 |
| cor(profileF,profileM) | -0.01 | 0.27 | -0.52 | 0.50 | 1.00 | 2185 | 1275 |
| cor(intF,enfaceF) | 0.04 | 0.27 | -0.51 | 0.54 | 1.00 | 3975 | 1587 |
| cor(intM,enfaceF) | -0.01 | 0.27 | -0.53 | 0.51 | 1.00 | 3710 | 1713 |
| cor(profileF,enfaceF) | -0.01 | 0.28 | -0.55 | 0.53 | 1.00 | 2413 | 1816 |
| cor(profileM,enfaceF) | 0.04 | 0.27 | -0.49 | 0.55 | 1.00 | 1974 | 1895 |
| cor(intF,enfaceM) | 0.05 | 0.26 | -0.43 | 0.54 | 1.00 | 3988 | 1623 |
| cor(intM,enfaceM) | 0.01 | 0.25 | -0.49 | 0.50 | 1.01 | 4079 | 1460 |
| cor(profileF,enfaceM) | 0.01 | 0.27 | -0.50 | 0.52 | 1.00 | 2987 | 1511 |
| cor(profileM,enfaceM) | -0.05 | 0.28 | -0.56 | 0.51 | 1.00 | 2034 | 1706 |
| cor(enfaceF,enfaceM) | -0.00 | 0.27 | -0.52 | 0.52 | 1.00 | 2465 | 1708 |
| cor(intF,bmiF) | 0.00 | 0.28 | -0.54 | 0.54 | 1.01 | 3708 | 1362 |
| cor(intM,bmiF) | -0.03 | 0.27 | -0.55 | 0.51 | 1.00 | 3809 | 1589 |
| cor(profileF,bmiF) | 0.01 | 0.27 | -0.56 | 0.53 | 1.00 | 2727 | 1348 |
| cor(profileM,bmiF) | -0.01 | 0.28 | -0.54 | 0.54 | 1.01 | 1914 | 1497 |
| cor(enfaceF,bmiF) | -0.01 | 0.28 | -0.54 | 0.52 | 1.00 | 1974 | 1583 |
| cor(enfaceM,bmiF) | 0.00 | 0.28 | -0.53 | 0.53 | 1.00 | 1818 | 1547 |
| cor(intF,bmiM) | 0.05 | 0.27 | -0.46 | 0.56 | 1.00 | 3620 | 1577 |
| cor(intM,bmiM) | 0.08 | 0.26 | -0.46 | 0.57 | 1.00 | 3633 | 1598 |
| cor(profileF,bmiM) | 0.01 | 0.27 | -0.52 | 0.53 | 1.00 | 2786 | 1822 |
| cor(profileM,bmiM) | -0.00 | 0.28 | -0.53 | 0.51 | 1.00 | 2290 | 1488 |
| cor(enfaceF,bmiM) | -0.01 | 0.28 | -0.53 | 0.51 | 1.00 | 1814 | 1933 |
| cor(enfaceM,bmiM) | -0.00 | 0.28 | -0.52 | 0.53 | 1.00 | 2000 | 1569 |
| cor(bmiF,bmiM) | 0.00 | 0.27 | -0.52 | 0.53 | 1.00 | 1440 | 1560 |
| cor(intF,ageF) | -0.02 | 0.24 | -0.46 | 0.43 | 1.00 | 2874 | 1669 |
| cor(intM,ageF) | -0.07 | 0.23 | -0.51 | 0.41 | 1.00 | 2679 | 1737 |
| cor(profileF,ageF) | 0.04 | 0.28 | -0.52 | 0.58 | 1.00 | 2018 | 1726 |
| cor(profileM,ageF) | 0.00 | 0.28 | -0.51 | 0.55 | 1.00 | 2025 | 1725 |
| cor(enfaceF,ageF) | -0.04 | 0.28 | -0.57 | 0.48 | 1.00 | 2112 | 1810 |
| cor(enfaceM,ageF) | 0.01 | 0.27 | -0.50 | 0.55 | 1.00 | 1301 | 1540 |
| cor(bmiF,ageF) | 0.02 | 0.27 | -0.51 | 0.55 | 1.00 | 1373 | 1700 |
| cor(bmiM,ageF) | -0.04 | 0.28 | -0.57 | 0.51 | 1.00 | 1547 | 1696 |
| cor(intF,ageM) | -0.03 | 0.23 | -0.48 | 0.42 | 1.00 | 3000 | 1624 |
| cor(intM,ageM) | -0.02 | 0.22 | -0.45 | 0.41 | 1.00 | 2756 | 1505 |
| cor(profileF,ageM) | 0.05 | 0.27 | -0.48 | 0.58 | 1.00 | 1282 | 1490 |
| cor(profileM,ageM) | -0.03 | 0.27 | -0.56 | 0.49 | 1.00 | 1434 | 1492 |
| cor(enfaceF,ageM) | -0.05 | 0.27 | -0.54 | 0.46 | 1.00 | 1324 | 1578 |
| cor(enfaceM,ageM) | 0.03 | 0.28 | -0.50 | 0.57 | 1.00 | 1551 | 1626 |
| cor(bmiF,ageM) | 0.01 | 0.28 | -0.52 | 0.53 | 1.01 | 1544 | 1727 |
| cor(bmiM,ageM) | 0.07 | 0.26 | -0.46 | 0.56 | 1.00 | 1437 | 1766 |
| cor(ageF,ageM) | 0.05 | 0.26 | -0.47 | 0.54 | 1.00 | 1672 | 1625 |
|  |  |  |  |  |  |  |  |
| **Regression Coefficients:** |  |  |  |  |  |  |  |
|  | **Estimate** | **Est.Error** | **l-95% CI** | **u-95% CI** | **Rhat** | **Bulk_ESS** | **Tail_ESS** |
| intF | 0.01 | 0.13 | -0.25 | 0.27 | 1.01 | 602 | 993 |
| intM | -0.02 | 0.14 | -0.30 | 0.24 | 1.01 | 817 | 1239 |
| profileF | 0.06 | 0.05 | -0.04 | 0.17 | 1.00 | 2824 | 1713 |
| profileM | -0.01 | 0.05 | -0.12 | 0.09 | 1.00 | 2800 | 1538 |
| enfaceF | -0.01 | 0.06 | -0.12 | 0.10 | 1.00 | 3209 | 1600 |
| enfaceM | 0.05 | 0.05 | -0.06 | 0.16 | 1.00 | 2615 | 1218 |
| bmiF | -0.01 | 0.04 | -0.08 | 0.06 | 1.00 | 3704 | 1576 |
| bmiM | -0.05 | 0.04 | -0.14 | 0.03 | 1.00 | 2939 | 1739 |
| ageF | -0.11 | 0.05 | -0.21 | -0.01 | 1.00 | 1848 | 1468 |
| ageM | -0.05 | 0.06 | -0.18 | 0.07 | 1.00 | 1879 | 1217 |
|  |  |  |  |  |  |  |  |
| **Further Distributional Parameters** | |  |  |  |  |  |  |
|  | **Estimate** | **Est.Error** | **l-95%CI** | **u-95%CI** | **Rhat** | **Bulk_ESS** | **Tail_ESS** |
| sigma | 0.83 | 0.02 | 0.80 | 0.86 | 1.00 | 4823 | 1424 |

**Table S7.** Multivariate multilevel Bayesian regression summary for four aggression components (PHAGR, VAGR, ANGER, HOST) with population-level random effects

| **Regression Coefficients:** |  |  |  |  |  |  |  |
| --- | --- | --- | --- | --- | --- | --- | --- |
|  | **Estimate** | **Est.Error** | **l-95% CI** | **u-95% CI** | **Rhat** | **Bulk_ESS** | **Tail_ESS** |
| PHAGR_intF | 0.01 | 0.10 | -0.18 | 0.20 | 1.00 | 4995 | 5831 |
| PHAGR_intM | 0.04 | 0.11 | -0.18 | 0.25 | 1.00 | 5226 | 6201 |
| PHAGR_profileF | 0.07 | 0.07 | -0.07 | 0.22 | 1.00 | 7210 | 5772 |
| PHAGR_profileM | 0.01 | 0.06 | -0.10 | 0.12 | 1.00 | 8931 | 6767 |
| PHAGR_enfaceF | -0.00 | 0.06 | -0.12 | 0.12 | 1.00 | 9339 | 6491 |
| PHAGR_enfaceM | -0.00 | 0.07 | -0.14 | 0.14 | 1.00 | 7476 | 6368 |
| PHAGR_bmiF | 0.00 | 0.04 | -0.08 | 0.08 | 1.00 | 11190 | 6797 |
| PHAGR_bmiM | -0.03 | 0.05 | -0.11 | 0.06 | 1.00 | 10637 | 6903 |
| PHAGR_ageF | -0.12 | 0.05 | -0.22 | -0.03 | 1.00 | 9832 | 6633 |
| PHAGR_ageM | -0.12 | 0.04 | -0.21 | -0.03 | 1.00 | 9553 | 6924 |
| VAGR_intF | 0.00 | 0.11 | -0.22 | 0.22 | 1.00 | 3246 | 4319 |
| VAGR_intM | -0.02 | 0.13 | -0.28 | 0.22 | 1.00 | 3720 | 5002 |
| VAGR_profileF | 0.05 | 0.06 | -0.06 | 0.17 | 1.00 | 8488 | 6798 |
| VAGR_profileM | -0.03 | 0.05 | -0.13 | 0.08 | 1.00 | 9020 | 6518 |
| VAGR_enfaceF | 0.02 | 0.07 | -0.10 | 0.15 | 1.00 | 7984 | 6200 |
| VAGR_enfaceM | 0.09 | 0.06 | -0.02 | 0.20 | 1.00 | 9587 | 6813 |
| VAGR_bmiF | 0.05 | 0.04 | -0.03 | 0.13 | 1.00 | 9979 | 6624 |
| VAGR_bmiM | -0.04 | 0.05 | -0.13 | 0.05 | 1.00 | 10750 | 7222 |
| VAGR_ageF | -0.10 | 0.05 | -0.19 | -0.01 | 1.00 | 9300 | 6211 |
| VAGR_ageM | -0.01 | 0.05 | -0.10 | 0.08 | 1.00 | 9055 | 6869 |
| ANGER_intF | 0.09 | 0.12 | -0.14 | 0.32 | 1.00 | 3050 | 4163 |
| ANGER_intM | -0.04 | 0.11 | -0.26 | 0.18 | 1.00 | 4126 | 5062 |
| ANGER_profileF | 0.07 | 0.05 | -0.03 | 0.17 | 1.00 | 9271 | 7075 |
| ANGER_profileM | 0.02 | 0.05 | -0.09 | 0.12 | 1.00 | 8120 | 6547 |
| ANGER_enfaceF | -0.01 | 0.05 | -0.12 | 0.09 | 1.00 | 9055 | 7026 |
| ANGER_enfaceM | 0.00 | 0.05 | -0.10 | 0.11 | 1.00 | 9306 | 6483 |
| ANGER_bmiF | -0.00 | 0.05 | -0.09 | 0.10 | 1.00 | 7425 | 6777 |
| ANGER_bmiM | -0.01 | 0.04 | -0.09 | 0.08 | 1.00 | 9922 | 6504 |
| ANGER_ageF | -0.11 | 0.04 | -0.19 | -0.02 | 1.00 | 9125 | 6875 |
| ANGER_ageM | -0.10 | 0.04 | -0.19 | -0.01 | 1.00 | 8395 | 6574 |
| HOST_intF | -0.05 | 0.14 | -0.32 | 0.21 | 1.00 | 2507 | 4209 |
| HOST_intM | -0.04 | 0.13 | -0.28 | 0.21 | 1.00 | 3532 | 4811 |
| HOST_profileF | 0.04 | 0.05 | -0.07 | 0.15 | 1.00 | 9017 | 6436 |
| HOST_profileM | -0.00 | 0.05 | -0.09 | 0.08 | 1.00 | 9973 | 6482 |
| HOST_enfaceF | -0.09 | 0.06 | -0.22 | 0.03 | 1.00 | 8209 | 6342 |
| HOST_enfaceM | 0.05 | 0.05 | -0.04 | 0.15 | 1.00 | 8757 | 6480 |
| HOST_bmiF | -0.03 | 0.04 | -0.10 | 0.04 | 1.00 | 10442 | 6658 |
| HOST_bmiM | -0.07 | 0.04 | -0.16 | 0.01 | 1.00 | 9621 | 6473 |
| HOST_ageF | -0.10 | 0.06 | -0.22 | 0.02 | 1.00 | 7091 | 6233 |
| HOST_ageM | -0.03 | 0.05 | -0.12 | 0.06 | 1.00 | 8385 | 6200 |
|  |  |  |  |  |  |  |  |
| **Further Distributional Parameters** | |  | **:** |  |  |  |  |
|  | **Estimate** | **Est.Error** | **l-95% CI** | **u-95% CI** | **Rhat** | **Bulk_ESS** | **Tail_ESS** |
| sigma_PHAGR | 0.91 | 0.02 | 0.87 | 0.94 1. | 0 | 10499 | 6240 |
| sigma_VAGR | 0.88 | 0.02 | 0.84 | 0.91 1. | 0 | 12099 | 6585 |
| sigma_ANGER | 0.88 | 0.02 | 0.85 | 0.91 1. | 0 | 10461 | 7254 |
| sigma_HOST | 0.81 | 0.02 | 0.78 | 0.84 1. | 0 | 10830 | 6257 |
|  |  |  |  |  |  |  |  |
| **Residual Correlations:** |  |  |  |  |  |  |  |
|  | **Estimate** | **Est.Error** | **l-95% CI** | **u-95% CI** | **Rhat** | **Bulk_ESS** | **Tail_ESS** |
| rescor(PHAGR,VAGR) | 0.41 | 0.02 | 0.36 | 0.45 | 1.00 | 10202 | 6279 |
| rescor(PHAGR,ANGER) | 0.51 | 0.02 | 0.47 | 0.55 | 1.00 | 9643 | 6787 |
| rescor(VAGR,ANGER) | 0.51 | 0.02 | 0.47 | 0.55 | 1.00 | 11546 | 7264 |
| rescor(PHAGR,HOST) | 0.41 | 0.02 | 0.37 | 0.46 | 1.00 | 10193 | 6614 |
| rescor(VAGR,HOST) | 0.45 | 0.02 | 0.41 | 0.50 | 1.00 | 11706 | 6718 |
| rescor(ANGER,HOST) | 0.52 | 0.02 | 0.48 | 0.56 | 1.00 | 12688 | 6610 |

**Section S6.** Total Aggression vs Administration procedure, sex, and country

**Figure S2**. Total Aggression scores, based on population and sex. Color of the dot and whisker correspond to the how the questionnaire was administered. Points correspond to sample means; whiskers reach the end of 95% SEM-based confidence interval. The plot, which is based on real data, suggests there are no systematic effects of the administration procedure.

| **Table S8.** Model predictions: DV is Total Aggressiveness. | | | | | | |
| --- | --- | --- | --- | --- | --- | --- |
| parameter | mean | sd | 2.5% CI | 97.5% CI | rhat | ess_bulk |
| a[1]: KON | 68.42 | 5.71 | 57.04 | 79.61 | 1.01 | 426.89 |
| a[2]: GAD | 63.10 | 5.67 | 51.91 | 74.25 | 1.01 | 484.47 |
| a[3]: THA | 63.86 | 6.01 | 52.11 | 75.42 | 1.01 | 568.32 |
| a[4]: CTR | 67.97 | 5.69 | 56.81 | 78.85 | 1.01 | 450.02 |
| a[5]: SRP | 66.62 | 5.97 | 54.23 | 78.01 | 1.00 | 585.79 |
| a[6]: LAD | 65.81 | 5.64 | 54.73 | 76.73 | 1.01 | 404.6 |
| a[7]: SIND | 68.34 | 5.56 | 57.33 | 79.14 | 1.01 | 400.98 |
| a[8]: CVST | 72.05 | 6.57 | 58.91 | 84.91 | 1.01 | 877.14 |
| a[9]: SIN | 61.50 | 5.50 | 50.54 | 72.05 | 1.01 | 377.82 |
| a[10]: TIB | 77.44 | 6.05 | 65.29 | 89.10 | 1.00 | 573.8 |
| a[11]: BEN | 73.20 | 5.57 | 62.30 | 83.92 | 1.01 | 402.02 |
| a[12]: KAL | 87.29 | 5.70 | 76.46 | 98.44 | 1.01 | 416.14 |
| a[13]: TA | 80.35 | 6.08 | 68.38 | 91.97 | 1.01 | 599.95 |
| a[14]: KAS | 78.02 | 5.55 | 66.97 | 88.53 | 1.01 | 377.14 |
| a[15]: TAM | 79.99 | 5.51 | 68.93 | 90.63 | 1.01 | 412.69 |
| a[16]: IND | 83.68 | 5.54 | 72.38 | 94.31 | 1.01 | 377.77 |
| a_bar: Intercept (across all) | 72.33 | 4.91 | 62.75 | 81.93 | 1.01 | 348.86 |
| b_sex: "male to female slope" | 0.68 | 0.84 | -1.02 | 2.26 | 1.00 | 2716.67 |
| b_assist[1]: Self-Administered | -0.92 | 6.52 | -13.75 | 11.96 | 1.01 | 904.95 |
| b_assist[2]: 10 %Assisted | 7.29 | 5.34 | -2.89 | 18.02 | 1.01 | 365.38 |
| b_assist[3]: 100 % Assisted | -6.25 | 5.52 | -17.05 | 4.62 | 1.01 | 444.62 |
| b_assist[4]: 50 % Assisted | -0.77 | 5.90 | -12.66 | 11.06 | 1.00 | 535.03 |
| b_assist[5]: 75 % Assisted | -0.74 | 5.92 | -12.37 | 10.60 | 1.01 | 581.04 |
| sigma_cult | 7.47 | 1.15 | 5.57 | 10.07 | 1.00 | 1960.64 |
| sigma | 16.38 | 0.29 | 15.83 | 16.98 | 1.00 | 3079.87 |

A simple Bayesian linear model reveals that effects of administration (unassisted or with different level of assistance) are unsystematic. Furthermore, the model predicts that some populations with variable levels of assistance yield similar Total Aggression scores and some populations with the same level of assistance score differently. All in all the, compared to administration, culture of the participant origin has much larger effects on Total Aggression.

**Table S9.** Summary of within- and between-population effects of aggression (W.TAGR, B.TAGR), age, and, BMI on frontal and profile facial shape.

| **model** | **predictor** | **SS** | **MS** | **R2** | **F** | **Z** | **p.value** |
| --- | --- | --- | --- | --- | --- | --- | --- |
| Enface–females | W.TAGR | 0.005 | 0.005 | 0.002 | 1.185 | 0.656 | 0.2590 |
|  | B.TAGR | 0.055 | 0.055 | 0.018 | 12.585 | 6.29 | 0.0010 |
|  | age | 0.069 | 0.069 | 0.022 | 15.673 | 6.656 | 0.0010 |
|  | BMI | 0.05 | 0.05 | 0.016 | 11.374 | 5.641 | 0.0010 |
| Enface–males | W.TAGR | 0.005 | 0.005 | 0.002 | 1.206 | 0.694 | 0.2580 |
|  | B.TAGR | 0.066 | 0.066 | 0.02 | 14.89 | 6.097 | 0.0010 |
|  | age | 0.088 | 0.088 | 0.027 | 19.919 | 7.417 | 0.0010 |
|  | BMI | 0.102 | 0.102 | 0.031 | 23 | 7.645 | 0.0010 |
| Profile–females | W.TAGR | 0.004 | 0.004 | 0.001 | 0.57 | -1.007 | 0.8430 |
|  | B.TAGR | 0.178 | 0.178 | 0.039 | 27.725 | 8.75 | 0.0010 |
|  | age | 0.103 | 0.103 | 0.022 | 16.042 | 8.097 | 0.0010 |
|  | BMI | 0.088 | 0.088 | 0.019 | 13.756 | 6.538 | 0.0010 |
| Profile–males | W.TAGR | 0.009 | 0.009 | 0.002 | 1.33 | 0.819 | 0.2080 |
|  | B.TAGR | 0.189 | 0.189 | 0.037 | 28.006 | 9.086 | 0.0010 |
|  | age | 0.156 | 0.156 | 0.031 | 23.129 | 7.41 | 0.0010 |
|  | BMI | 0.099 | 0.099 | 0.019 | 14.68 | 6.473 | 0.0010 |
